# Estimating the Effect of Intimate Partner Violence on Women’s Use of Contraception: A Systematic Review and Meta-Analysis

**DOI:** 10.1371/journal.pone.0118234

**Published:** 2015-02-18

**Authors:** Lauren Maxwell, Karen Devries, Danielle Zionts, Jeanne L. Alhusen, Jacquelyn Campbell

**Affiliations:** 1 Department of Epidemiology, Biostatistics, & Occupational Health, McGill University, Montréal, Québec, Canada; 2 Social and Mathematical Epidemiology Group and Gender Violence and Health Centre, Department of Global Health and Development, London School of Hygiene and Tropical Medicine, London, United Kingdom; 3 Department of Community-Public Health, Johns Hopkins University School of Nursing, Baltimore, Maryland, United States of America; University of Rochester, UNITED STATES

## Abstract

**Background:**

Intimate partner violence (IPV) is an important global public health problem. While there is a growing literature on the association between IPV and women’s reproductive health (RH) outcomes, most studies are cross-sectional—which weakens inference about the causal effect of IPV on women’s RH. This systematic review synthesizes existing evidence from the strongest study designs to estimate the impact of IPV on women’s use of contraception.

**Methods:**

We searched 11 electronic databases from January of 1980 to 3 December 2013 and reviewed reference lists from systematic reviews for studies examining IPV and contraceptive use. To be able to infer causality, we limited our review to studies that had longitudinal measures of either IPV or women’s use of contraception.

**Results:**

Of the 1,574 articles identified by the search, we included 179 articles in the full text review and extracted data from 12 studies that met our inclusion criteria. We limited the meta-analysis to seven studies that could be classified as subject to low or moderate levels of bias. Women’s experience of IPV was associated with a significant reduction in the odds of using contraception (n = 14,866; OR: 0.47; 95% CI: 0.25, 0.85; *I^2^* = 92%; 95% CI*_I_^2^*: 87%, 96%). Restricting to studies that measured the effect of IPV on women’s use of partner dependent contraceptive methods was associated with a reduction in the heterogeneity of the overall estimate. In the three studies that examined women’s likelihood of using male condoms with their partners, experience of IPV was associated with a significant decrease in condom use (OR: 0.48; 95% CI_OR_: 0.32, 0.72; *I^2^* = 51%; 95% CI*_I_^2^*: 0%, 86%).

**Conclusions:**

IPV is associated with a reduction in women’s use of contraception; women who experience IPV are less likely to report using condoms with their male partners. Family planning and HIV prevention programs should consider women’s experiences of IPV.

## Introduction

Intimate partner violence (IPV) is both a human rights issue and an important public health concern. IPV is the most prevalent form of gender-based violence; the 2013 Global Burden of Disease Study estimates that 30% of women age 15 or over have experienced physical or sexual IPV [1]. Cross-sectional studies from a number of countries indicate that IPV is associated with a constellation of women’s reproductive health (RH) outcomes related to contraceptive use including rapid repeat pregnancy (pregnancy within 24 months of a previous pregnancy) [2–4]; unintended pregnancy [5–7]; pregnancy termination [7–9]; and incident HIV infection [9–13].

Reproductive coercion, taking control of women’s RH, is one form of IPV. Women may be forced to have sex or to practice unprotected sex by their male partners and male partners may sabotage women’s use of family planning (FP) to increase their female partner’s dependency or to otherwise express their control over their partner’s decision making [5,6,14–18]. Qualitative and cross-sectional studies suggest that birth control sabotage is a type of reproductive coercion and that women may adopt contraceptive methods that they can hide from their partners or that do not require negotiation with their male partners to mitigate this barrier [16,19,20].

Women’s ability to control the timing, spacing, and number of their pregnancies is a critical health and human rights issue. Addressing the unmet need for FP is a key step to meeting Millennium Development Goals (MDGs) 3, 4 and 5 which aim to promote gender equity; reduce maternal and child mortality; and ensure universal access to RH including contraception and antenatal care, respectively. Understanding how IPV modifies women’s ability to adopt contraception is central to designing FP interventions that allow women who experience IPV to manage their fertility and to informing HIV prevention interventions.

### Purpose of the Review

In this review we attempt to estimate the causal effect of IPV on contraceptive use. Most of the existing literature on the association between women’s experience of IPV and contraceptive use is based on estimates of associations and does not address issues of temporality, which restricts our ability to infer the causal effect of IPV on women’s contraceptive use. This study builds on recent systematic reviews that have found an association between IPV and different sexual health outcomes. A 2014 systematic review found an association between IPV and termination of pregnancy, but included all study designs, including cross-sectional studies [8]. A 2007 systematic review provided an overview of studies that estimated the association between physical IPV and women’s sexual health outcomes, including contraceptive use and pregnancy termination, but included all study designs and did not include a meta-analysis of included studies [21]. To understand the scope of existing evidence for the effect of IPV on women’s use of contraception, we restrict this systematic review to studies with longitudinal measures of IPV and/or contraceptive use and to studies that use a case-control design. In keeping with a prior systematic review related to IPV, we define a longitudinal study as one where either the exposure or the outcome was measured at a minimum of two time points [22].

## Methods

### Search Strategy

We searched 11 biomedical databases: PubMed (Medline); OvidSP (EMBASE, PsycINFO, CINAHL); Global Health Library (including LILACS, AFRO, EMRO, PAHO, WHOLIS, WPRO); and POPLINE from 1 January 1980 to 3 December 2013 to identify research studies on IPV and women’s RH outcomes. Because of the changes in women’s access to and knowledge of contraceptive methods over time with the introduction of novel contraceptive methods such as the IUD and oral contraceptives, we restricted our search to 1980 onwards. In addition to the electronic database searches, we identified citations by reviewing reference lists from relevant reviews and studies. We adapted Medical Subject Headings (MeSH) and text-based search terms for IPV from prior peer-reviewed literature and systematic reviews of intimate partner violence [23,24]. An information scientist reviewed the search strategy and pilot tested the search in Medline, EMBASE and PsycINFO with one of the authors (LM). The PubMed (Medline) search strategy is provided in S1 Supplementary Text.

### Inclusion Criteria

We included studies of women and girls of any age that evaluated the association between respondents’ exposure to IPV by a male partner and an outcome related to women’s RH or to infant health in the initial review of titles and abstracts identified in our search. We included all types of IPV, whether the abuse was classified as physical, sexual, psychological, or economic. Because we identified more studies than expected with longitudinal measures of IPV and RH outcomes, we restricted our inclusion criteria to studies with an outcome related to women’s use of contraception subsequent to the initial search. We did not restrict our definition of contraception and included articles with modern contraceptive methods and traditional methods of FP. We did not restrict studies by geographic location or language of publication.

### Study Designs

While we did not use study design filters in our search, we only considered longitudinal (panel, cohort, randomized controlled trails (RCTs)) or case-control studies for inclusion in the systematic review. Where the intervention arm of an RCT intervened on both the exposure to IPV and outcome of contraceptive use, we used the effect measure from the control arm of the RCT.

### Screening and Data Extraction

We followed the Cochrane guidelines for the systematic review of non-randomized studies and for the review of randomized studies [25,26], and prepared the manuscript using PRISMA guidelines (See S1 Supplementary Table for the PRISMA checklist). We registered our systematic review protocol with the international prospective register of systematic reviews, PROSPERO, (registration number CRD42013006457, S2 Supplementary Text) before initiating our search. One reviewer (LM) conducted the search; removed duplicate studies; and identified additional studies through a review of related articles and systematic reviews. In keeping with the Cochrane recommendations for the systematic review of non-randomized studies, we assessed study design features, rather than study design labels, to evaluate whether studies could be classified as longitudinal or case-control study designs [26]. Two reviewers (LM and DZ) independently screened the titles and abstracts of all studies identified through the search strategy to identify studies that met the inclusion criteria for full text review. In cases of disagreement about study inclusion, the study was included in the full text review.

For studies that met our inclusion criteria, but where the study authors did not include a relevant effect measure, we contacted the authors for additional information or for participant-level data. For studies that included a definition of sexual or physical violence that was not limited to violence perpetrated by a male intimate partner, we contacted the authors to determine whether they had measured intimate partner-specific violence. For studies that were in languages other than English, French, or Spanish, an epidemiologist fluent in the language of the study worked with LM to determine whether the study was relevant for inclusion. Data were extracted independently by LM and DZ using a standardized, pre-piloted electronic data extraction form. Discrepancies in data extraction between assessors were resolved through consensus.

We extracted data on study location; design; sample characteristics and size; type of violence; interview method and setting; the scale used to measure IPV; IPV-specific interviewer training; confounders; outcome definitions; and effect measures.

### Quality Assessment

We assessed the potential for bias within each study using the Cochrane Methodological Quality Assessment of Observational Studies for longitudinal and case-control studies (S3 Supplementary Text); the Cochrane Risk of Bias Tool for RCTs (S4 Supplementary Text); and a series of questions specific to assessing bias in measures of IPV exposure [26]. Two reviewers (LM, DZ) independently assessed each study’s potential for being affected by the most common forms of bias in observational studies: selection, confounding, performance, detection, and attrition bias. In consultation with other subject matter experts, we created a directed acyclic graph (DAG) to differentiate between important confounders and variables that are likely on the causal pathway between IPV and contraceptive use (S6 Fig.). DAGs are used to encode *a priori* causal knowledge to identify and distinguish between the variables that are likely to be common causes of the exposure and outcome (confounders) and the variables that are on the causal pathway between the exposure and outcome (mediators) [27–29]. Focusing on the assessment of selection and confounding bias and temporal ordering of cause and effect, we classified studies as having a low, moderate, or high probability of bias. Studies were classified as having low probability of bias if they were not obviously affected by the aforementioned sources of bias and if they used methods to reduce the probability of measured confounding in non-randomized studies such as propensity score matching. Studies classified as subject to moderate bias adjusted for relevant confounders and had no obvious sources of bias.

### Data Analysis

We used random effects meta-analysis to estimate the pooled odds ratio (OR) for the association between IPV and women’s use of contraception across studies. We chose random rather than fixed effects models because we expected a high level of heterogeneity across studies and standard errors estimated using random effects models are generally more conservative than those estimated with fixed effects models [30]. All studies reported ORs adjusted for confounding. We used the adjusted OR from each study in the meta-analysis. Where necessary, we inverted the adjusted OR presented in the published article so that all effect measures in the meta-analysis were operating in the same direction (contraceptive use versus non-use). All analysis were done using Stata version 13.0 (StataCorp LP, College Station Texas). While we calculated a pooled OR across all studies regardless of study quality, we restricted additional meta-analysis to studies that were classified as having a moderate or low probability of bias in keeping with the Cochrane recommendations for the synthesis of data from observational studies [26].

We used the *I* ^2^ statistic to assess heterogeneity of effect estimates across included studies. The *I* ^2^ statistic quantifies the proportion of variation across studies due to actual variation rather than chance [31]. Based on the Cochrane Handbook for Systematic Reviews’ recommendations, we interpreted an *I* ^2^ statistic between 0% and 40% as representing an insignificant amount of heterogeneity; 30% to 60% as moderate heterogeneity; and 50% to 90% as substantial heterogeneity; and 75% to 100% as considerable heterogeneity [32]. We estimated the uncertainty in the heterogeneity estimate using Stata’s *heterogi* command [33] and used Stata’s *metaninf* command to estimate the influence of each individual study on the pooled estimate [34]. We assessed the degree of probable publication bias by reviewing funnel plots that compare log ORs to their standard errors. We used Egger’s test of funnel plot asymmetry to test the null hypothesis of no small-study effects.

### Ethics Statement

Because the data included in this analysis contain no identifying information and are publically available, ethical approval was not required for this review.

## Results

### Overview of Selected Studies

**Fig 1:**
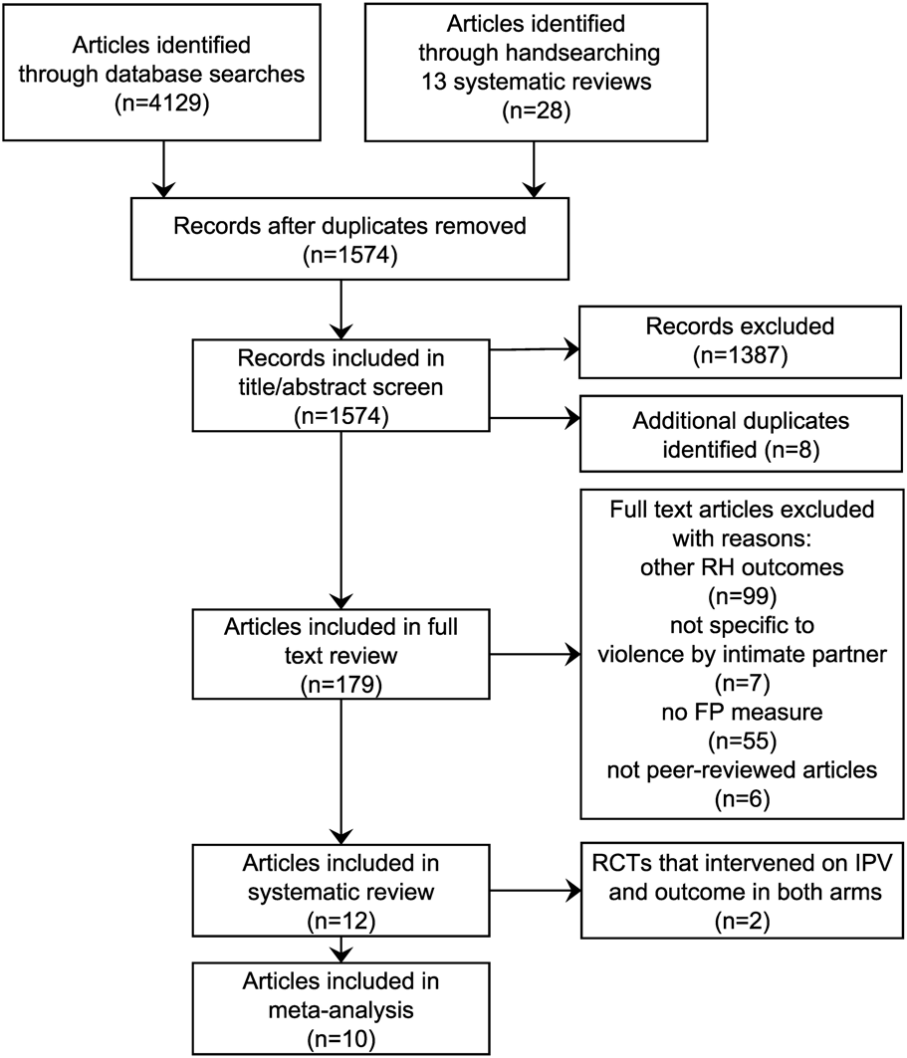
PRISMA flow diagram.

Fig. 1 presents the PRISMA flow diagram of the study selection process. We reviewed the titles and abstracts of 1,574 articles and included 179 articles in the full text review. Twelve studies with 17,827 participants met our inclusion criteria (please refer to S2 Supplementary Table for a list of articles excluded following full text review). We excluded effect estimates from two RCTs that intervened on the exposure and the outcome in both the control and intervention arms (n = 385) [35,36]. Table 1 presents a detailed overview of the case-control, the control arm of the included RCT, and the eight other longitudinal studies included in the systematic review. The intervention and control arms of both the included and excluded RCTs are summarized in S3 Supplementary Table. All of the studies were fairly recent: the earliest publication date was 2005 and five of the 10 studies were published after 2010. Six of the 10 studies were conducted in the US [37–42]; two studies were conducted in Central and South America [43,44]; one study was from India [45]; and the RCT was conducted in Africa [46]. The studies included a diversity of populations: two studies only included adolescents [40,41] and one study was limited to methadone clinic patients [37]. Three studies restricted their study populations to ever-pregnant or ever-delivered women. One study was limited to women who were pregnant at baseline and had delivered prior to follow-up [43]; and two studies included women who had given birth within the year prior to their baseline interview [39,41]. Five of the studies included clinic or hospital-based populations [37,38,41,42,44]; three studies included a subset of participants in a population-level longitudinal study [40,43,45]. The size of studies ranged from 225 to 6,414 (IQR = 337–2,058) subjects. While the median follow-up time was one year, the time between interviews ranged from three months [44] to five years [45].

**Table 1:** Summary of longitudinal, RCT, and case-control studies that examine the association between IPV and women’s contraceptive use.

Author year	Population, country	Intimate partner	Exposure	Comparison	Outcome	OR (95% CI)
El-Bassel 2005 [37]	337 participants aged 18–55 in a methadone maintenance program who had a male intimate partner at 14 clinics; NYC, US	Sexual or dating relationship during past year with boyfriend, spouse, regular sex partner, children’s father	Physical or sexual IPV in last 6 months	Neither physical nor sexual IPV during the last 6 months	Always vs sometimes/never use of condoms in last 6 months	0.41 (0.24, 0.71)
Fantasia 2012 [38]	2000 WRA (mean age 22) who received RH services at 4 clinics. Excluded women who reported history of violence as a child who did not report current IPV; US	A male partner	Physical, sexual, or emotional IPV during past year	Never experienced physical, sexual, emotional IPV	Not using vs using contraception in past year^a^	9.7 (6.2, 15.2)
McCarraher2006 [44]	293 women who came to a clinic for oral contraceptives; Bolivia	NR	Ever experience of FP-related physical, or emotional IPV^b^	Never experienced FP-related physical or emotional IPV	Discontinuation vs continuation of oral contraceptive use in last 3–6 months	1.91 (1.00, 3.66)
Kacaneck 2013^c^ [46]	Subset of MIRA trial [76] participants ages 18–49 recruited from clinics. Limited to participants who answered questions on IPV at baseline and 12 month follow-up; South Africa, Zimbabwe	Person you most often had sex with in the last 3 months	Physical or sexual IPV in past year^d^	No physical or sexual IPV in last year	Sometimes/never vs always use of condoms in last 3 months	1.66 (1.39, 1.98)
Salazar 2011 [43]	398 delivered women (median age 27) who participated in the Health and Demographic Surveillance System; Nicaragua	A male partner	Physical, sexual, or emotional IPV during year prior to follow-up	No physical, sexual, or emotional IPV during year prior to follow-up	Current use vs nonuse of reversible contraception 40–47 months after delivery^e^	2.59 (1.24, 5.40)
Scribano 2013 [39]	6414 delivered women/girls ages 13–45 who participated in an in-home intervention for low-income, first-time mothers; US	Husband, ex-husband, boyfriend, or ex-boyfriend	Physical IPV in year prior to baseline (one year after delivery)	No physical IPV in year prior to baseline	Current use vs nonuse of contraception one year after baseline^f^	0.34 (p = 0.001)^g^
Stephenson 2013 [45]	4111 rural women/girls ages 15–39 who participated in 2 waves of National Family Health Survey, restricted to currently married women who are not sterilized and not using contraception at baseline; India	Husband	Physical IPV in year prior to baseline	No physical IPV in year prior to baseline	Use vs nonuse of modern contraception between baseline and follow-up^h^	0.73 (0.55, 0.96)
Teitelman 2008 [40]	2058 adolescents aged 11–26 who participated in Wave III of the AddHealth Study, reported at least one male partner in the past year, reported previous or currently sexual activity; US	One of up to three special romantic relationships with a male within the 18 months prior to Wave III interview	Physical or emotional IPV in the year prior to Wave III interview	Neither physical nor emotional IPV in the year prior to Wave III interview	Sometimes/never vs always use of condoms in year prior to Wave III interview	1.59 (1.16, 2.18)
Van Horne 2009 [41]	632 adolescents aged 15–19 who gave birth at a hospital and complete both the 6 and 12 month surveys; US	Current or previous boyfriend or husband	Physical IPV in 6 months prior to baseline	No physical IPV in 6 months prior to baseline	Sometimes vs always use of condoms one year after delivery^i^	3.82 (1.4, 10.6)
Williams 2008^k^ [42]	225 hospital-based participants aged 18–50 who had a male partner in past year and who had not had a tubal ligation or hysterectomy; US	Married to, living with, or involved with a male partner	Physical or emotional IPV in year prior to baseline	No physical or emotional IPV in year prior to baseline	Nonuse vs use of contraception in year prior to baseline^j^	1.8 (0.7, 4.8)

OR, adjusted odds ratio; CI, confidence interval; NYC, New York City; US, United States; IPV, intimate partner violence; WRA, women of reproductive age; FP, family planning; NR, not reported

^a^Contraception includes: injectables, implants, IUD, male and female sterilization, oral contraceptives, contraceptive patch and ring, barrier methods, withdrawal and “natural methods.”

^b^Types of method-related partner violence include: partner became angry with the woman for using a contraceptive method; the partner threatened the woman because she was using a method; the partner hit the woman for using a method; and the woman was afraid that her partner would hit her because she was using a method.

^c^Control arm of randomized controlled trial.

^d^While the Kacanek study includes a more expansive definition of IPV that includes physical, sexual, emotional violence and fear of violence; we use the estimate that includes physical and sexual IPV for comparability given that no other study includes “fear of violence” as a form of IPV.

^e^Reversible contraception excludes sterilization and includes oral contraceptives, IUD, injection, condom, calendar-rhythm method, and withdrawal.

^f^Article does not define which contraceptive methods included; personal communication from author, contraception includes: oral contraceptives, IUD, injectables, condoms, and spermicide.

^g^No CI or standard error reported.

^h^Modern contraception includes: birth control pills, IUD, injection, condom, and male or female sterilization.

^i^Also report never versus always use of condoms at follow-up: OR: 3.17 (95% CI: 1.1, 8.9).

^j^Case-control study.

^k^Contraception includes: injectables; implants; IUD; birth control pills; condom; diaphragm or cervical cap; foam, jelly, or cream; female condom, vaginal pouch; emergency contraception; “natural family planning” such as the rhythm method, safe period by calendar, safe period by temperature, or cervical mucus test; withdrawal or pulling-out. Respondents who report female or male sterilization were excluded from the analysis.

### Study Quality

Table 2 provides details on the quality of the one case-control and nine longitudinal studies (n = 17,442) that were included in the meta-analysis. We classified the estimate from the control arm of the RCT as having a low probability of bias [46]. Five of the other longitudinal studies were classified as subject to moderate bias [37–39,43,45]; two longitudinal studies [40,44] and the case-control study [42] were determined to have a high level of bias.

**Table 2:** Quality assessment of included case-control, RCT, and longitudinal studies.

Author year	Selection bias	Confounding bias	Temporality	Performance bias	Detection bias	Attrition bias	Overall likelihood of bias	Qualitative assessment
El-Bassel 2005 [37]	Moderate (75% of those screened participated. Those sampled and enrolled possibly different from those sampled and not enrolled in ways related to the outcome)	Low; *adjusted for*: demographic factors; prior non-partner abuse; mental health; incarceration; drug use; relationship dependencies^a^; baseline condom use. Uses matching	Low	Low (in person interview, used CTS2)	Low (usage in last 6 months)	Low (MI; sensitivity analysis indicates informative censoring unlikely)	Moderate	Special population limits generalizability; adjustment for factors on causal pathway (social support, depression, PTSD) may bias effect estimate towards the null
Fantasia 2012 [38]	Low (retrospective chart review)	Moderate; *adjusted for*: demographic factors; STIs	Low	Moderate (interview part of routine medical care)	Low (current usage at follow-up)	Low (retrospective chart review)	Moderate	Definition of contraception includes non-modern methods; could have adjusted for income to control for access to contraceptive methods
Kacanek^b^ 2013 [46]	Moderate (clinic based population; restrict to participants who answered IPV-related questions at baseline and 12-month follow-up)	Low; *adjusted for confounders that were not balanced after randomization*; program site; partner absence; partner HIV status; partner drug use	Low	Low (audio-computer assisted interview)	Low (usage in last 3 months)	Low (sample restricted to those who complete both interviews)	Low	Control arm of RCT; authors followed randomization procedures; restricting to women who completed both baseline and follow-up interviews may introduce selection bias if IPV is related to contraceptive use and women who experience IPV are more likely to drop out of the study
McCarrah-er 2006 [44]	Moderate (excluded transient women, may have missed most vulnerable women)	High; *adjusted for*; partner education, experience of side effects of birth control pills	High (concurrent exposure and outcome measures)	High (in person interview, limited exposure definition to contraceptive method related violence)	Low (current usage at follow-up)	High (53% of those selected from clinic records not reached for follow-up)	High	Restricting definition to method-related IPV limits inference; no adjustment for demographic factors
Salazar 2011 [43]	Low (women were randomly selected from a Demographic Surveillance System)	Moderate; *adjusted for*: maternal age, education; household SES, urban/rural location; parity	Low	Low (in-person interview, used CTS)	Low (current usage at follow-up)	Low (sensitivity analysis indicates informative censoring unlikely)	Moderate	Definition of contraception includes non-modern methods
Scribano 2013 [39]	Low (includes all eligible clients enrolled in Nurse Family Partnership Program)	Moderate; *adjusted for*: confounder categories: demographic and IPV-related confounders (no description of actual confounders)	Low	Low (in person report to RN; use AAS)	Low (current usage at follow-up)	NR	Moderate	Study uses data from an intensive home visiting program that could have affected exposure and outcome
Stephens-on 2013 [45]	Moderate (restricted to rural, married women not using contraceptives at baseline; if exposure associated with contraceptive use, may have selected a higher proportion of exposed individuals who had outcome)	Moderate; *adjusted for*: maternal age, education; household SES; partner education; parity; state; spousal age difference; fertility intentions.	Low	Low (in person interview; IPV measure based on CTS2 used for DHS IPV measures)	Low (current usage at follow-up)	Low (sensitivity analysis indicates informative censoring unlikely)	Moderate	3–5 years between measures complicates inference; restricting to contraceptive nonusers at baseline may introduce selection bias if IPV affects contraceptive use; adjustment for factor on causal pathway (parity) may bias effect estimate towards the null
Teitelman 2008 [40]	Low (includes all adolescents who report having a male intimate partner)	Moderate; *adjusted for*: maternal age, minority group (no adjustment for access to contraception)	High (concurrent exposure and outcome measures)	Low (in person interview; used CTS)	Moderate (IPV status measured at same time as outcome)	Low (sensitivity analysis indicates informative censoring unlikely)	High	Did not report the temporally ordered effect estimate (instead measures IPV and contraceptive use at Wave III)
Van Horne 2009 [41]	Moderate (restricted to those who completed baseline and follow-up; those retained had higher depression levels and lower self-esteem than non- retained)	Moderate; *adjusted for*: minority group, church attendance, health system monitoring, partner refusing condom use, condom use at 6 months, pregnancy intent	Low	Low (mail survey; modified AAS)	Low (use in last 6 months)	Low (sample restricted to those who complete both interviews)	Moderate	Potential selection bias; adjustment for factor on causal pathway (partner refusal to wear condoms) likely biases effect estimate towards the null
Williams^c^ 2008 [42]	High (distraught women not screened; 62% of those approached didn’t participate; different selection method for cases and controls)	High; *adjusted for*: maternal age, relationship status (does not adjust for factors that could influence access to contraception (SES))	NA	High (women complete survey regardless of partner presence; used CTS2, SVAWS, WEB)	Low (use in last 6 months)	NR	High	Definition of contraception includes non-modern methods; probable selection, confounding, and performance bias

CTS2, modified Conflict Tactics Scale; MI, multiple imputation; PTSD, post-traumatic stress disorder; STI, sexually transmitted infection; IPV, intimate partner violence; CTS, Conflict Tactics Scale; HIV, human immunodeficiency virus; SES, socio economic status; RN, registered nurse; AAS, Abuse Assessment Screen; NR, not reported; DHS, Demographic and Health Surveys; NA, not applicable; SVAWS, Severity of Violence Against Women Scale; WEB, Women’s Experience with Battering Scale.

^a^Relationship dependencies include: financial dependencies (housing dependency contribution to the household) and drug dependency (partner paid for the woman’s drugs), whether a partner had ever become angry with them, threatened them, hit them, or if they feared their partner would hit them because they were using a contraceptive method.

^b^Control arm of randomized controlled trial.

^c^Case-control study.

**IPV measurement**. Studies varied in their description of IPV and in the time period over which they assessed IPV. Definitions of intimate partner ranged from “person you most often had sex with in the last 3 months” [46] to “husband” [45]. Seven of the 10 studies reported using a validated scale to measure women’s report of violence. The Conflict Tactics Scale was used in five studies [37,40,41,43,45]; two studies reported using the Abuse Assessment Screen [39,42]; one study used the CTS, in addition to the Severity of Violence Against Women Scale and the Women’s Experience with Battering Scale. Researchers generally classify IPV into four separate categories: emotional, physical, sexual, and economic. Physical violence was the most widely measured exposure; all studies included physical violence in their exposure definition; four studies only measured the effect of physical violence [39,41,42,45]. Different forms of IPV are not necessarily correlated [1] so studies that limited their classification of IPV to physical IPV may have underestimated the total effect of IPV. In two studies that included multiple categorizations of IPV (physical, sexual, and emotional) [42,43], we used the effect estimates from the most expansive definition to better understand the effect of IPV on women’s use of contraception. In one study with multiple categorizations of IPV, we used the classification that included physical and sexual IPV rather than the classification that included physical, sexual, emotional, and fear of IPV because this was the only study to include fear of IPV as a type of IPV [46].

**Contraceptive use measurement**. Five of the 10 studies limited their estimate of the effect of IPV on women’s contraceptive use to one or two methods, including oral contraceptives [44], condoms and diaphragms [46], and condoms [37,40,41]. Three of the five studies that included a number of different methods considered withdrawal and the rhythm method as contraceptive methods [38,42,43], while the remaining two studies limited their definition to modern methods of contraception. Women were asked to recall their contraceptive use during the last year [38,40,42], last six months [37,41] or to report current use [39,43]. Effect estimates included comparisons between use and non-use in seven studies [38,39,42–46], and always and sometimes or never use in three studies [37,40,41]. The three studies that included women who had given birth measured women’s use of contraception one [41], two [39], and four [43] years after delivery, when women are unlikely to continue to use lactational amenorrhea for FP [47]. We inverted the ORs from six of the studies in Table 1 [38,40–42,44,46] to facilitate the comparison of outcome measures (e.g., contraceptive use versus non-use) across studies.

**Confounder adjustment**. All studies reported effect measures that were adjusted for basic demographic confounders including maternal age, socioeconomic status, and minority status. Studies also adjusted for prior experience of non-partner abuse [37]; mental health [37]; fertility intentions [41,45]; and relationship status [42], amongst other potential confounders. Some studies adjusted for factors thought to mediate the relationship between IPV and contraceptive use including parity [45], partner refusal to wear condoms [41], social support, and depression [37].

**Additional outcomes**. Four studies provided effect estimates for additional RH outcomes, including: the likelihood of using a hidden method of contraception [38,44]; the odds of using emergency contraception [38]; reporting multiple sex partners [40]; shortened interpartum intervals [39] and the likelihood of engaging in unprotected anal sex [37]. We only extracted data on measures of contraceptive use to allow for a comparison across studies.

### Meta-analysis

This review aims to evaluate and synthesize existing evidence for the effect of IPV on women’s use of contraception. As described *a priori* in our research protocol, we restricted our overall meta-analysis to studies that were classified as having low or moderate levels of bias and only included the three studies that were classified as having a high level of bias in the subgroup analysis of the association between the level of bias and the estimated pooled OR.

### Subgroup Analysis

**Fig 2:**
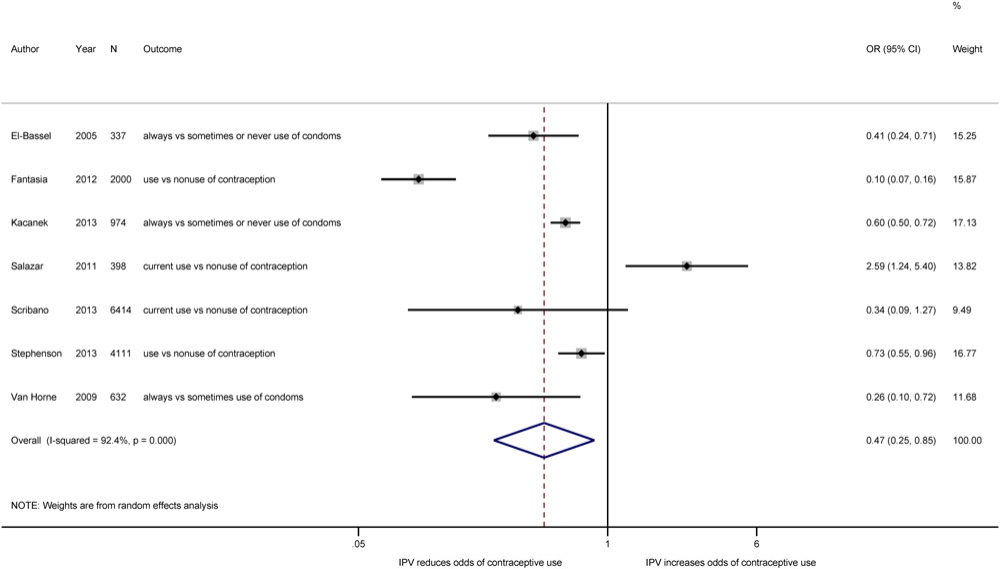
Meta-analysis of the association between IPV and contraceptive use. Estimated effect measures from the Fantasia, Kacanek, and Van Horne studies have been inverted to present estimates of contraceptive use rather than non-use.

As indicated both in the forest plot of the seven studies classified having low or moderate levels of bias (Fig. 2) and in the forest plot that includes all 10 studies irrespective of bias (S1 Fig.), there is considerable heterogeneity in effect measures across studies. We performed a series of subgroup analysis to explore potential sources of heterogeneity. We planned to conduct subgroup analysis on study quality, type of IPV, and whether studies were limited to pregnant versus non-pregnant participants *a priori*. We included a subgroup analysis comparing studies with non-modern forms of FP and modern contraception *post hoc* after identifying several studies that included natural methods of FP, like the rhythm method and withdrawal, rather than restricting to modern contraceptive methods.

**Fig 3:**
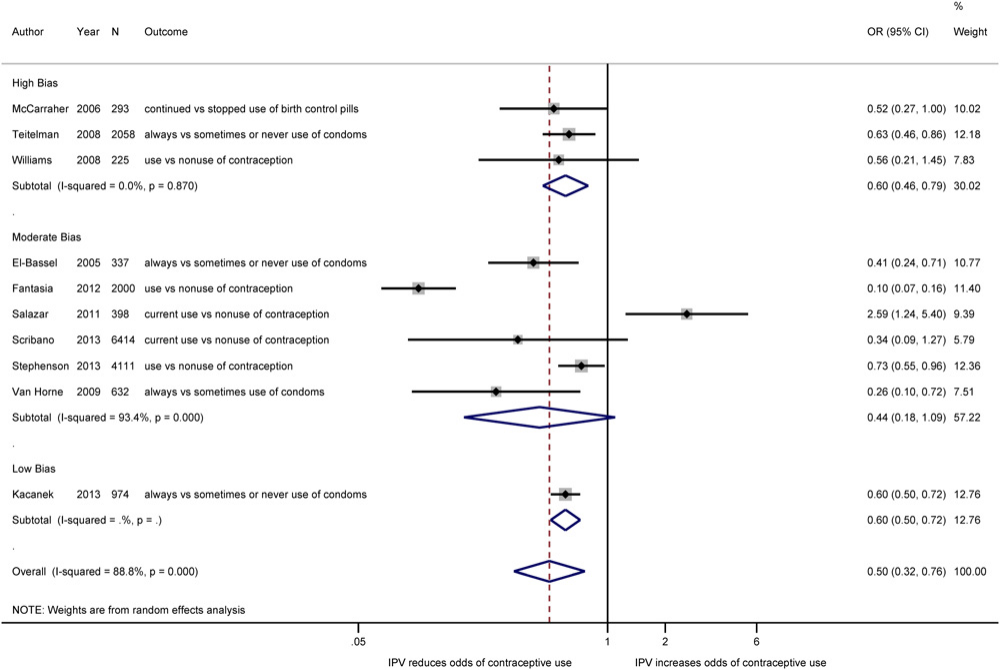
Meta-analysis of the association between IPV and contraceptive use, by level of bias. Estimated effect measures from the Fantasia, McCarraher, Kacanek, Teitelman, Van Horne, and Williams studies have been inverted to present estimates of contraceptive use rather than non-use.

**Methodological quality**. In Fig. 3, we show the changes in the effect estimates by level of probable bias. Of the 10 included studies (n = 17,442), three studies were classified as having a high probability of bias; six studies were found to have a moderate level of bias; and one study, the control arm of the included RCT, was classified as having a low probability of bias. Grouping studies according to their probable bias did not explain a significant amount of the heterogeneity in the overall estimate (*I* ^2^ = 89%, 95% CI_*I*_^*2*^: 81%, 93%). Both studies classified as having a high probability of bias and studies classified as subject to low or moderate levels of bias indicated that IPV was associated with a decrease in women’s odds of using contraception (OR_high bias_: 0.60; 95% CI_OR_: 0.46, 0.79; *I* ^2^ = 0%, 95% CI_*I*_^*2*^: 0%, 90%; OR_moderate_: 0.44; 95% CI_OR_: 0.18, 1.09; *I* ^2^_moderate_ = 93%, 95% CI_*I*_^*2*^: 88%, 96%; and OR_low_: 0.60; 95% CI_OR_: 0.50, 0.72 respectively). While we extracted data from two longitudinal studies where the exposure was not clearly measured before the outcome and the case-control study which had different selection procedures for cases and controls (selection bias), we classified these studies as having a high probability of bias and excluded them from subsequent subgroup and meta-analysis.

**Fig 4:**
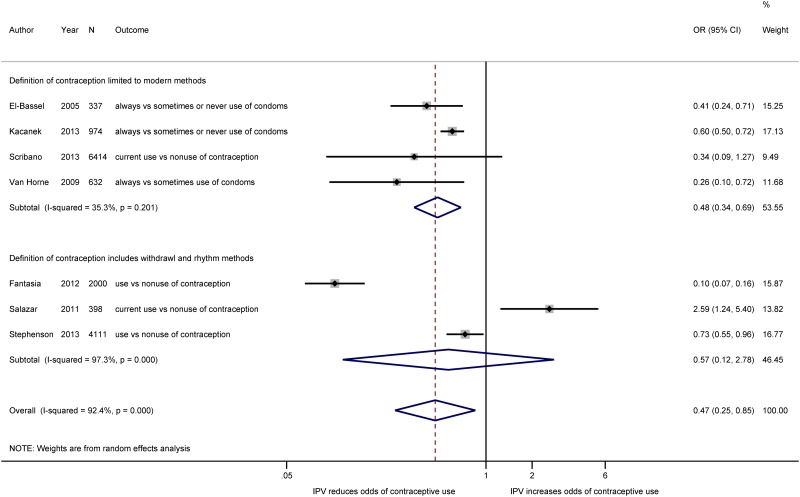
Meta-analysis of the association between IPV and contraceptive use, by definition of FP. Estimated effect measures from the Fantasia, Kacanek, and Van Horne studies have been inverted to present estimates of contraceptive use rather than non-use.

**Classification of contraceptive methods**. In Fig. 4, we compare the pooled effect measures by whether studies included withdrawal and the rhythm method in their classification of contraceptive methods or restricted their definition to modern contraceptive methods (e.g., injectables, oral contraceptives, IUD, condoms, bilateral tubal ligation). As mentioned previously, we only included effect estimates from the seven studies classified as having a low or moderate probability of bias (n = 14,866). The three studies whose definition of contraception ranged from modern contraception to less effective forms of FP such as withdrawal and the rhythm method [38,42,43], were more likely to find a non-significant association between IPV and women’s use of contraception (n = 6,509; OR: 0.57; 95% CI_OR_: 0.12, 2.78; *I* ^2^ = 97%, 95% CI_*I*_^*2*^: 95%, 99%) than the four studies that estimated the effect of IPV on women’s use of modern contraceptive methods (n = 8,357; OR: 0.48; 95% CI_OR_: 0.34, 0.69; *I* ^2^ = 35%, 95% CI_*I*_^*2*^: 0%, 77%). Excluding estimates from studies that included the rhythm method and “natural family planning methods” and limiting inference to the effect of IPV on women’s use modern contraceptive methods reduced the heterogeneity in the pooled estimate from 97% to 35%. The high level of heterogeneity in the pooled estimate from studies that included natural FP methods remained after removing the Salazar study (*I* ^2^ = 98%). The difference in heterogeneity when comparing studies whose definition of contraception includes withdrawal and the rhythm method to studies that limit inference to modern methods may indicate that the broad definition of contraception in these studies is a source of heterogeneity in the overall meta-analysis. In the three studies that measured women’s reports of their male partner’s condom use, (S1 Fig.) women who experienced IPV were significantly less likely to report that their partners used condoms than women who did not experience IPV (n = 1,943; OR: 0.48; 95% CI_OR_: 0.32, 0.72; *I* ^*2*^ = 51%, 95% CI_*I*_^*2*^: 0%, 86%).

**Type of IPV**. We used the estimated effect of physical IPV from the two studies that included multiple classifications of IPV [43,46] in a meta-analysis of the five studies that measured exposure to physical IPV, presented in S2 Fig. Physical IPV was significantly associated with a reduction in women’s use of contraception in four of the five studies, although the pooled OR was not statistically significant and was subject to a high degree of heterogeneity (n = 12,529; OR: 0.71; 95% CI_OR_: 0.45, 1.12; *I* ^*2*^ = 79%, 95% CI_*I*_^*2*^: 50%, 91%). Because there were only two studies that specifically explored the effect of sexual or emotional IPV on women’s use of contraception [43,46], we could not perform separate meta-analysis of these exposures.

**Study population**. In S3 Fig., we compare the pooled effect measures between studies that did and did not restrict to women who had given birth. The pooled effect measure for the three studies that restricted to delivered women was not significantly different from that of the four studies that did not restrict to delivered women and did not did not help to explain the heterogeneity in the overall estimate (OR_restricted to delivered women_: 0.64; 95% CI_OR_: 0.13, 3.25; *I* ^*2*^ = 87%, 95% CI_*I*_^*2*^: 64%, 96%; OR_not restricted to delivered women_: 0.38; 95% CI_OR_: 0.19, 0.76; *I* ^*2*^ = 94%, 95% CI_*I*_^*2*^: 90%, 97%).

### Overview of Data Synthesis

The results of the meta-analysis, wherein we pooled the ORs from the 10 studies included in the meta-analysis irrespective of study quality, indicated that women who experience IPV have a lower odds of adopting contraception than women who do not experience IPV, although the results were subject to a high level of heterogeneity (n = 17,442; OR: 0.50; 95% CI_OR_: 0.32, 0.76; *I* ^*2*^ = 89%, OR_*I*_^*2*^: 81%, 93%). When we restricted our inference to the seven studies classified as subject to low or moderate bias, the odds of women who had experienced IPV reporting contraceptive use were 53% lower than the odds for women who had not experienced IPV (n = 14,866; 95% CI_OR_: 0.25, 0.85; *I* ^*2*^ = 92%, 95% CI_*I*_^*2*^: 87%, 96%). While no single study was found to unduly influence the pooled OR, not all of the effect estimates operated in the same direction. In six of the seven studies included in the pooled meta-analysis, where we restricted to studies classified as subject to moderate or low bias, women who experienced IPV had a lower odds of reporting contraceptive use than women who did not. The Salazar study [43], which examined the effect of physical, sexual, and emotional IPV on the use of a contraceptive method other than sterilization was a notable exception: the odds of women who experienced IPV using contraception were 2.59 times the odds of women who did not experience IPV at either baseline or follow-up (95% CI: 1.24, 5.40). The difference between the effect estimates in the Salazar study and in the other six studies may be caused by country-level differences in access to contraception and norms surrounding contraceptive use. The Nicaraguan government works to ensure open access to contraception through FP programs in the public and private health sector and uses community-based distribution of contraception to reach underserved communities [48,49]. Nicaragua’s prevalence of contraceptive use, estimated at 73% in 2013, is well above the global average for contraceptive use of 63% [50]. While exclusion of the Salazar study from the pooled estimate did decrease the point estimate and increase the precision of the pooled OR, removal of the Salazar study did not lead to a decrease the degree of heterogeneity in the pooled estimate of studies with low and moderate bias (OR_without Salazar_: 0.36; 95% CI_OR_: 0.20, 0.65; *I* ^*2*^ = 92%, 95% CI_*I*_^*2*^: 85%, 96%).

### Analysis of Violence Severity

**Fig 5:**
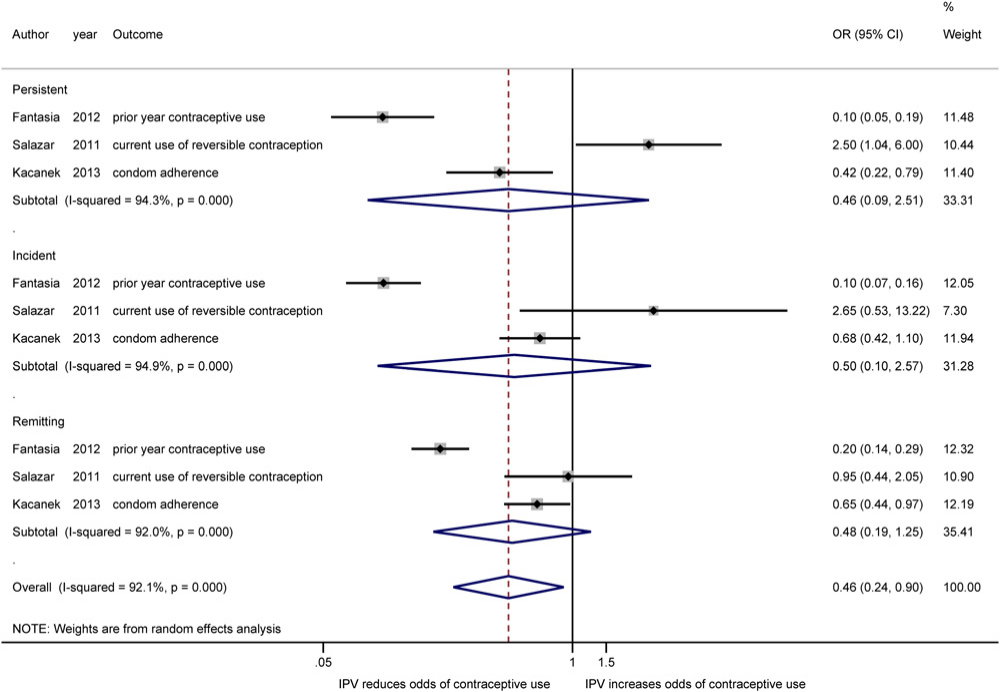
Meta-analysis of the association between IPV and contraceptive use, by duration of exposure. Estimated effect measures from the Fantasia and Kacanek studies have been inverted to present estimates of contraceptive use rather than non-use.

There are different ways of conceptualizing the severity of violence. Some classifications use the type of violence; others examine the frequency of violent acts. Three of the longitudinal studies in this review conceptualized violence severity using the duration of reported violence [38,43,46]. For studies that included several measures of violence duration, we used the measure that most closely matched the duration of violence reported in other studies in the violence severity meta-analysis. In Table 3, we report the effect estimates for the three studies that included measures of the duration of violence. Categorizing violence as either moderate or severe by using the type of violence has been used previously to predict the severity of adverse health outcomes [51,52]. Using the classification of violence severity from the Kacanek article [46], these three studies’ classification of each participant’s experience of IPV as persistent, measured at both time points; incident, only measured at the most recent time; or remitting, not measured at the most recent time, but measured previously, provide an opportunity to explore how the duration of IPV can differentially affect contraceptive use. Fig. 5 presents a forest plot of these duration measures.

**Table 3:** Association between the duration of intimate partner violence and women’s contraceptive use as compared to women who have no history of intimate partner violence.

Author year	Study type	Persistent		Incident		Remitting		Comparison group	Outcome measure
		Definition	OR (95% CI)	Definition	OR (95% CI)	Definition	OR (95% CI)		
Fantasia 2012^a^ [38]	retrospective cohort	Past year IPV and IPV during the past 5 years	9.8 (5.3, 18.3)	Past year IPV only	9.7 (6.2, 15.2)	No past year IPV but a history of IPV	4.9 (3.5, 7.0)	Never having experienced physical, sexual, or emotional IPV	Not using contraception^b^
Salazar 2011 [43]	panel	Past year IPV and IPV during last pregnancy	2.50 (1.04, 5.99)	Past year IPV, no IPV during last pregnancy	2.65 (0.53, 13.2)	IPV during last pregnancy, no IPV in past year	0.95 (0.44, 2.04)	No IPV during last pregnancy, no IPV in year prior to interview	Current use of contraception
Kacanek 2013 [46]	RCT	Physical or sexual IPV present at baseline and at follow-up	2.4 (1.25, 4.50)	Physical or sexual IPV not present at baseline, is present at follow-up	1.48 (0.91, 2.40)	Physical or sexual IPV present at baseline, not present at follow-up	1.53 (1.04, 2.30)	No physical or sexual IPV at baseline or at follow-up	Sometimes/never vs always use of condoms in last 3 months

OR, adjusted odds ratio; CI, confidence interval; RCT, randomized controlled trial.

^a^Includes an additional measure of IPV duration, excluded here for comparability: past year IPV and IPV extending for greater than 5 years, OR: 7.7 (95%CI: 3.3, 17.6).

^b^Contraception includes: injectables, implants, IUD, male and female sterilization, oral contraceptives, contraceptive patch and ring, barrier methods, withdrawal and “natural methods.”

In contrast to the estimated effect of IPV on women’s use of contraception in the other two studies, the estimates from the Salazar study [43] indicate that women who experience IPV are more likely to adopt contraception than women who do not. Across all three studies that include measures of IPV duration, the magnitude of the effect of IPV on women’s use (or non-use) of contraception is greatest for women who experience persistent IPV.

## Discussion

### Summary of Main Findings

Results of the meta-analysis suggest that IPV affects women’s use of contraception. Author’s classification of contraceptive methods was a potential source of heterogeneity. There is some evidence for a dose-response relationship between duration of IPV exposure and women’s contraceptive use. IPV is associated with a decrease in women’s use of partner dependent methods; women who experienced IPV were less likely to report that their male partners used condoms than women who did not. The association between IPV and contraceptive use is likely modified by contextual factors. In Nicaragua, for example, open access to contraceptive methods and the wide cultural acceptability of contraception may mean that women who experience IPV are more likely to use contraception than women who do not in the Nicaraguan context.

### Limitations of Included Studies

While the objective of this review was to identify and synthesize the best available estimates of the effect of IPV on women’s use of contraception, the studies that we identified were subject to a number of limitations that could have biased our assessment of the relationship between IPV and women’s use of contraception.

**Misclassification of the exposure**. Three of the studies limited their definition of IPV to physical violence. Exposure to different forms of IPV is not necessarily correlated [1]; limiting the definition of IPV may underestimate the total effect of IPV which would bias effect estimates towards the null value if IPV affect contraceptive use. As expected, the magnitude of effect estimates from studies that included multiple forms of IPV was greater than the magnitude of effect estimates from studies that only measured one form of IPV. The range in the definition of intimate partners across studies may have led to underreporting of violence in studies that did not consider previous partners or sexual partners as intimate partners. This misclassification could have also biased results towards the null value.

All exposure and outcome measures were based on self-reports. Under-reporting of IPV is both difficult to evaluate and a pervasive issue in IPV-related research [53]. Women who report IPV may experience an increase in IPV incidence or severity [54–56]. Women may be unlikely both because of the fear of increased violence and because of the stigma associated with reporting [54,57]. Studies that do not interview participants alone or that use interviewers who have not been trained in interviewing survivors of IPV likely lead to higher levels of under-reporting of the exposure than studies that follow the WHO best practices for interviewing women about IPV [9]. No included studies described their interviewer training practices.

**Misclassification of the outcome**. Three of the 10 studies included in the systematic review used expansive definitions of contraception that included withdrawal, “natural methods,” and/or the rhythm method [38,42,43]. Women who report using withdrawal or the rhythm method may not experience the same barriers to access and complicated negotiations with their partners and family members faced by women who try to use modern contraceptive methods. The classification of women who are not using a modern method of contraception as using contraception could have biased the association between IPV and women’s adoption of contraception towards the null value in these three studies.

**Potential selection bias**. Studies that restrict inference to participants who are retained in each survey wave may introduce selection bias if participants who are retained differ from those who are not in ways that are related to the exposure and to the outcome of interest [58]. In one study that restricted to participants who were retained in both survey waves, researchers found that retained participants had lower levels of self-efficacy and higher levels of depression than those who were not retained [41]. The three studies that restricted their populations to women who had given birth [39,41,43] may have introduced selection bias by selecting a lower percentage of women who experience IPV than that of the target population given that women who experience IPV may be more likely to terminate their pregnancies than women who do not [8].

### Strengths and Limitations of This Review

This is the first systematic review to provide a quantitative estimate of the relationship between IPV and contraceptive use. We did not restrict our review by language and included a diversity of databases. Two researchers independently screened all articles and extracted data from research studies included in the systematic review. For studies that included a measure of gender based violence that was not limited to IPV, we contacted authors to see if they could provide us with an effect estimate limited to IPV.

In addition to the aforementioned limitations of individual studies that may underestimate the association between IPV and contraceptive use, there are several limitations to this analysis. All of the studies included in the systematic review estimate the odds of contraceptive uptake. Odds ratios estimated using logistic regression generally overestimate relative risks for prevalent outcomes [59], like contraceptive use, so the pooled ORs likely overestimate the magnitude of the relative probability of using contraception when comparing women who do and do not experience IPV. Because the pooled estimates include studies with different measures of IPV and the type and duration of contraceptive use, the pooled estimates are meant to provide an approximate estimate of the average association between IPV and contraceptive use and should be interpreted with caution.

Although contraceptive method type seemed to account for some heterogeneity in the overall estimate, statistical heterogeneity is not necessarily reflective of clinically important heterogeneity [60]. While the test for heterogeneity of the OR of included studies was not significant when restricting to studies that restricted inference to modern contraception, the statistical test for heterogeneity is underpowered and may not distinguish between actual and chance heterogeneity [60,61]. Given the limited number of studies included in the meta-analysis, we were not able to use meta-regression to explore sources of heterogeneity in the overall estimate [32,62,63].

Because researchers are unlikely to publish research protocols for observational studies, the results presented here are likely subject to publication bias. The heterogeneity of our exposure and outcome and the small number of included studies necessarily complicates the assessment of publication bias [60]. We assessed the degree of probable publication bias visually by using a funnel plot to compare study log ORs with their standard errors for all 10 studies included in the overall meta-analysis (S4 Fig.) and for the seven studies classified as having a low or moderate probability of bias (S5 Fig.). With few studies it is difficult to assess potential publication bias, but both contour funnel plots used to evaluate publication bias are missing studies from the area of non-significance, which suggests the possibility of some publication bias. Similarly, Egger’s test of funnel plot asymmetry indicated that we could not reject the null hypothesis of no small-study effects when considering all 10 studies (*p* = 0.764) or when limiting inference to the seven studies classified as having a low or moderate probability of bias (*p* = 0.640) [64].

### Causal Inference

Temporality, consistency, strength of association, biological plausibility, and dose-response are necessary components of the classical approach to causal inference [65] and are used to evaluate the strength of evidence from meta-analysis of observational studies [66]. In all studies included in the meta-analysis, the measurement of the exposure proceeded the outcome. The estimates of the effect of IPV on women’s use of contraception are relatively strong and consistent across a diversity of populations in four countries, with the exception of the estimate from the Nicaraguan study as noted previously. A prior systematic review provides a comprehensive overview of the direct and indirect pathways through which IPV may affect women’s contraceptive use [21]. Pre-existing research in a number of countries suggests that women change their contraceptive behavior in the face of IPV and that IPV affects women’s ability to negotiate condom use with their male partners [5,15,19,67,68]. The meta-analysis of the three studies that examined violence severity suggests a dose-response relationship between women’s exposure to IPV and their use of contraception. The results from this review suggest that IPV may have a causal effect on women’s use of contraception and may affect women’s use of male condoms with their partners. The suggestion that IPV may have a causal effect on women’s use of contraception must be evaluated with the understanding that this is a meta-analysis of effect estimates from observational studies. While we limited the meta-analysis to studies classified as having a low or moderate probability of bias, the findings of our meta-analysis are subject to the residual confounding that likely affects effect estimates from included studies [69]. In the absence of individual patient level data, as would be used in an individual patient data meta-analysis, we cannot be sure that the decrease in contraceptive use across studies is caused by women’s experience of IPV and not by miss-measured or unmeasured confounders.

### Implications for Future Research

The purpose of this review was to summarize existing evidence from longitudinal and case-control studies for the effect of IPV on women’s use of contraception. Of the nine studies that included longitudinal measures, two studies could not be used to estimate the effect of IPV on women’s use of contraception because the exposure and the outcome were measured contemporaneously. There is a clear need for additional research where the temporal ordering of exposure and outcome allow for a better understanding of the causal effect of IPV on women’s use of contraception.

Two of the seven studies included in the meta-analysis only assessed the effect of physical violence on women’s use of contraception. Future research should include a more complete definition of IPV to better estimate the total impact of IPV on women’s use of contraception. Sexual and physical IPV may have differential effects on women’s use of contraception; additional research should consider including estimates of the association between different forms of IPV and contraceptive use. Reproductive coercion may affect women’s contraceptive use in the absence of other forms of IPV. Future research could explore additional causal pathways through which reproductive coercion, community norms about violence, gender inequality, and the influence of in-laws interact with other forms of IPV to affect women’s contraceptive use.

This review indicates that women who experience IPV are less likely to report that their male partners use condoms than women who do not. Future research might examine the impact of harm reduction strategies on the ability of women who experience IPV to use condoms with their male partners. Condom use requires a complex set of negotiations between a woman and her male partner. Prior research indicates that women who experience IPV may be more likely to adopt contraceptive methods that they can hide from their partners [14,70,71]. Further research is needed to understand whether women who experience IPV prefer to adopt long-acting reversible and permanent contraceptive methods that are less likely to require their partner’s involvement. In meta-analysis, sources of heterogeneity may indicate important sources of bias [72]. This review suggests that contraceptive method type should be considered as a source of bias in estimates of the effect of IPV on women’s contraceptive use. To best understand how IPV modifies women’s adoption of contraception or the type of method that women choose to adopt, future research should consider limiting inference to modern methods of contraception and might differentiate between methods that do and do not require ongoing negotiations between a woman and her male partner.

Across studies that included a classification of IPV duration, the magnitude of the effect of persistent IPV on women’s use of contraception was greater than that of remittent or incident IPV. Future research could continue to explore the importance of IPV duration in predicting women’s use of contraception.

### Implications for Practice

Understanding how IPV modifies women’s ability to adopt or to continue to use contraception is central to informing evidence-based FP and HIV prevention interventions. Prior research indicates that IPV is associated with HIV infection [10–12]. Given that this systematic review found that women who report IPV are less likely to use condoms than women who do not report IPV, HIV prevention interventions should consider addressing IPV.

The WHO recommends that providers ask women about exposure to IPV when assessing conditions that may be caused or complicated by IPV [73] and FP providers should consider asking specifically about reproductive coercion when screening for IPV [18,74]. The American College of Obstetricians and Gynecologists offers specific guidance for providers to ask women about their experience of reproductive coercion [74]. Recent clinical guidelines suggest that health care providers caring for women who experience reproductive coercion should offer contraceptive methods that are less susceptible to partner sabotage (e.g., IUD and implant) while counseling women about IPV and safety planning strategies [75]. Ensuring that women can access long-acting and permanent contraceptive methods could help women who experience IPV plan their families.

## Conclusion

Pooling estimates from seven longitudinal studies that included 14,866 participants, women who experienced IPV were less likely to use contraception than women who did not. The high level of heterogeneity in the pooled estimate is typical of systematic reviews of non-randomized studies and may be due to the inclusion of heterogeneous populations and exposure and outcome measures or to residual confounding of effect measures from individual studies [26]. Future research that includes longitudinal measures of IPV and women’s use of modern contraceptive methods is needed to better understand the impact of IPV on women’s adoption of contraception.

## Supporting Information

S1 Fig Meta-analysis of the association between IPV and condom use. (PDF)

S2 Fig Meta-analysis of the association between physical partner violence and contraceptive use. (PDF)

S3 Fig Meta-analysis of the association between IPV and contraceptive use, by whether study restricted to delivered women. (PDF)

S4 Fig Funnel plot to assess publication bias. (PDF)

S5 Fig Funnel plot to assess publication bias in seven studies classified as having a low or moderate probability of bias. (PDF)

S6 Fig Directed acyclic graph of hypothesized causal relationship between IPV and women’s contraceptive use. (PDF)

S1 Table PRISMA Checklist. (PDF)

S2 Table List of articles excluded after full text review with reasons for exclusion. (PDF)

S3 Table Summary of randomized controlled trials that examine the association between IPV and women contraceptive use. (PDF)

S1 Text Ovid (Medline) search strategy. (PDF)

S2 Text Systematic review protocol. (PDF)

S3 Text Cochrane Methodological Quality Assessment of Observational Studies for Longitudinal and Case Control studies. (PDF)

S4 Text Cochrane Risk of Bias Tool for Randomized Controlled Trials. (PDF)
