# Supplementary material for: Estimating the Effect of Intimate Partner Violence on Women’s Use of Contraception: A Systematic Review and Meta-Analysis
Source: PLoS One. 2015 Feb 18;10(2):e0118234. doi: 10.1371/journal.pone.0118234 (PMC4334227; doi:10.1371/journal.pone.0118234)
Supplement: S2 Fig — (PDF) [file pone.0118234.s002.pdf]

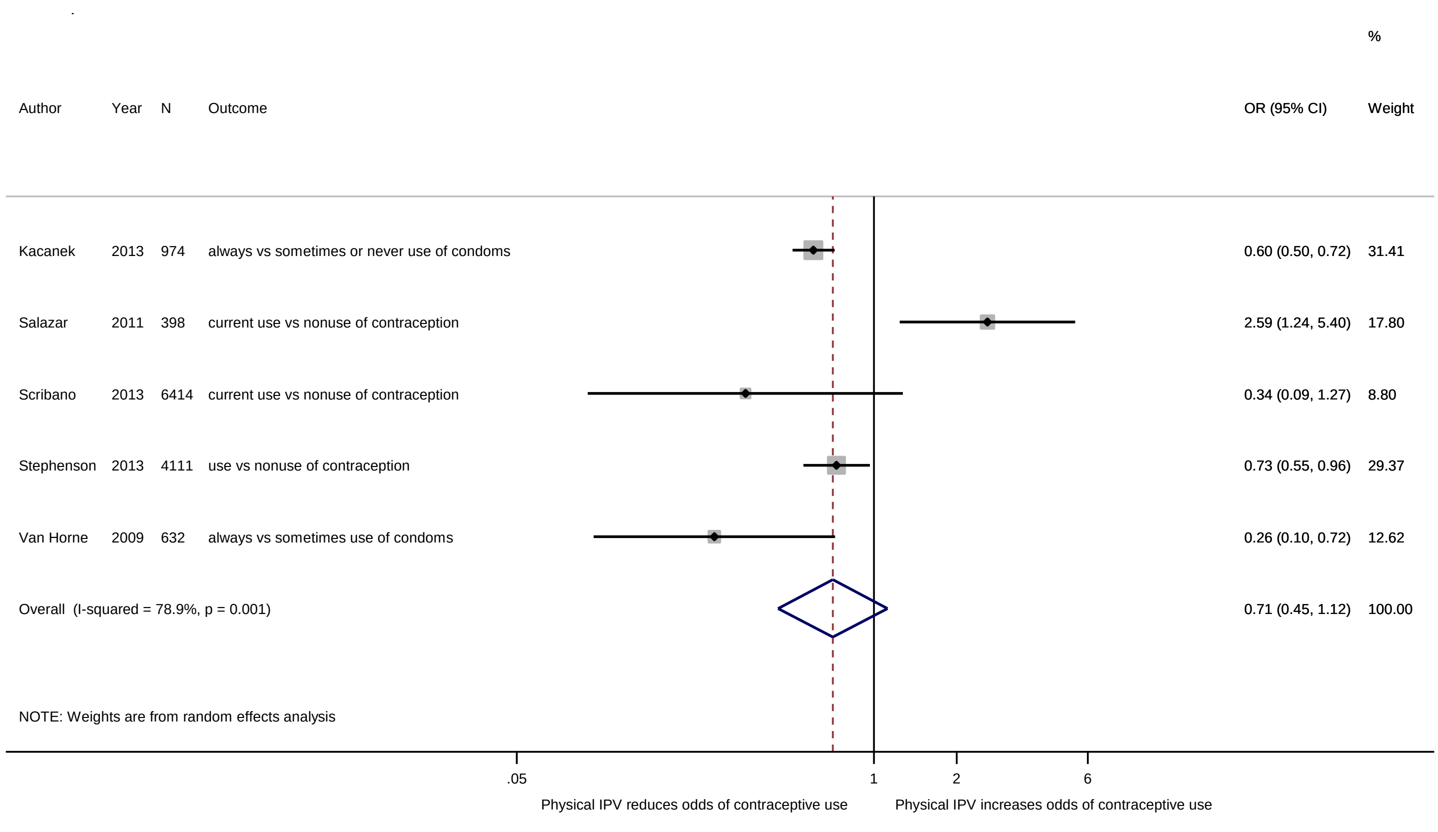

Figure S2. Meta-analysis of the association between physical partner violence and contraceptive use. Estimated effect measures from the Kacanek and Van Horne studies have been inverted to present estimates of contraceptive use rather than non-use.
