# Supplementary material for: Estimating the Effect of Intimate Partner Violence on Women’s Use of Contraception: A Systematic Review and Meta-Analysis
Source: PLoS One. 2015 Feb 18;10(2):e0118234. doi: 10.1371/journal.pone.0118234 (PMC4334227; doi:10.1371/journal.pone.0118234)
Supplement: S3 Fig — (PDF) [file pone.0118234.s003.pdf]

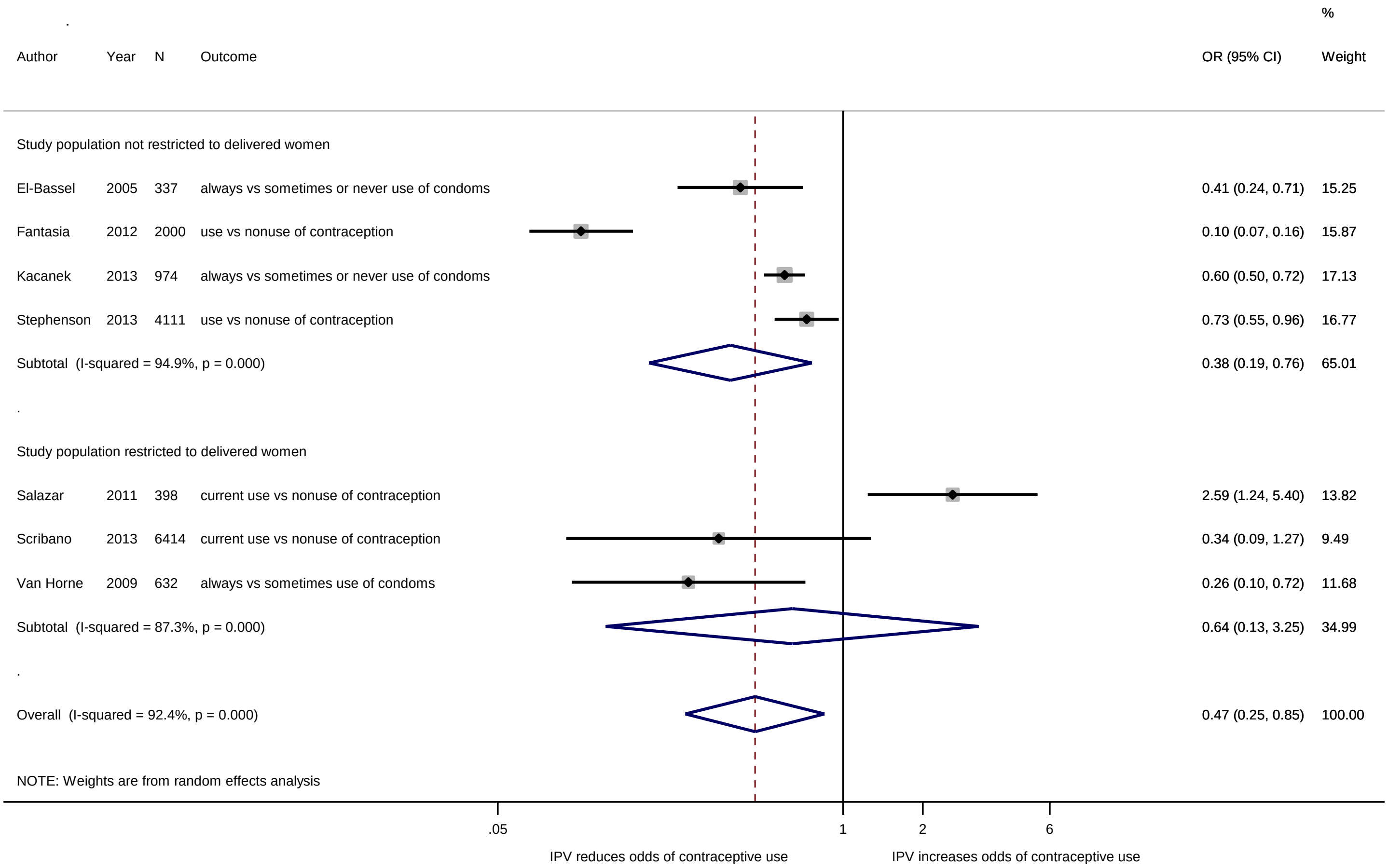

Figure S3. Meta-analysis of the association between IPV and contraceptive use, by whether study restricted to delivered women. Estimated effect measures from the Fantasia, Kacanek, and Van Horne studies have been inverted to present estimates of contraceptive use rather than non-use.
