# Supplementary material for: Estimating the Effect of Intimate Partner Violence on Women’s Use of Contraception: A Systematic Review and Meta-Analysis
Source: PLoS One. 2015 Feb 18;10(2):e0118234. doi: 10.1371/journal.pone.0118234 (PMC4334227; doi:10.1371/journal.pone.0118234)
Supplement: S6 Fig — (PDF) [file pone.0118234.s006.pdf]

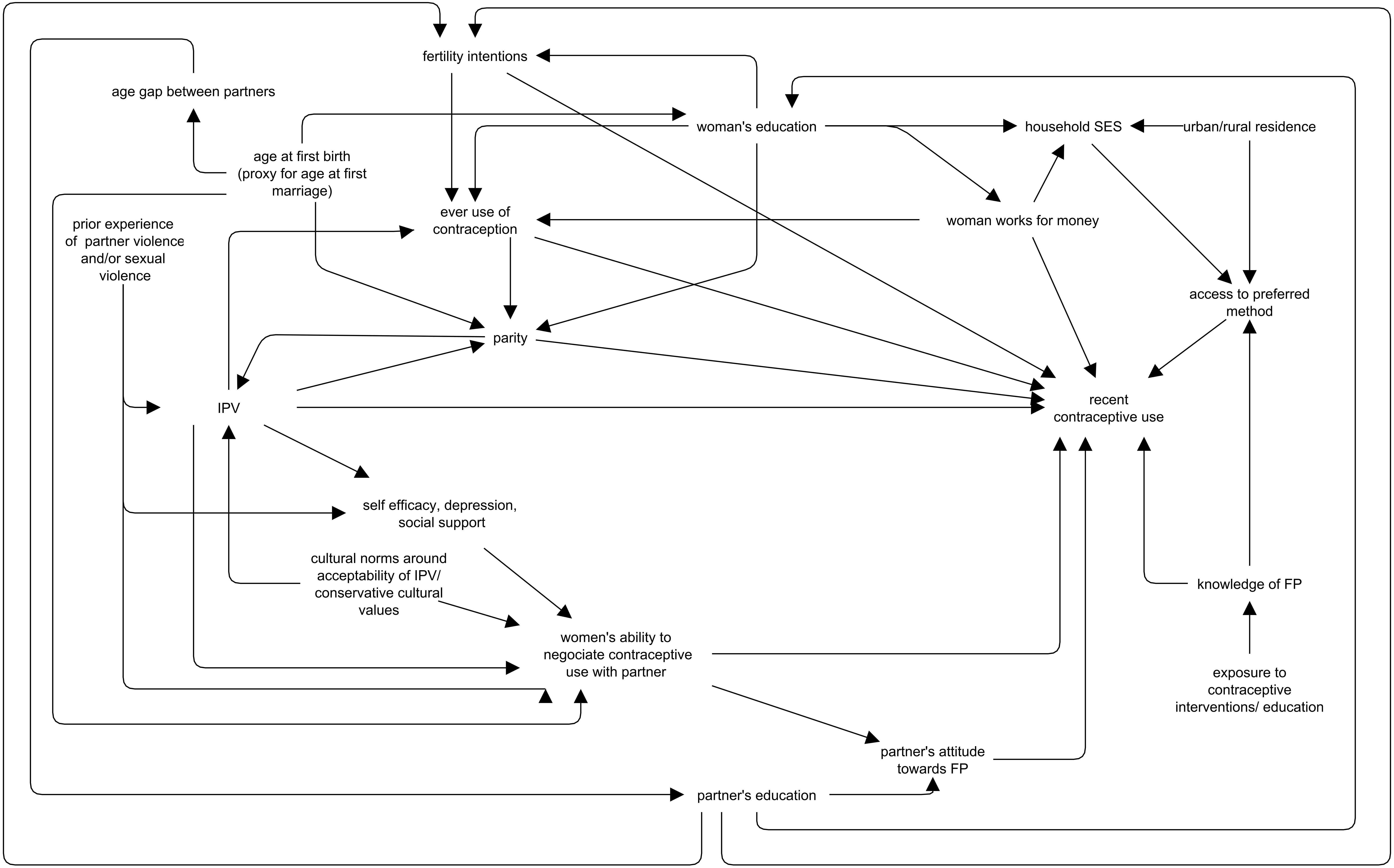

Figure S6. Directed acyclic graph of hypothesized causal relationship between IPV and women's contraceptive use
