# Supplementary material for: Estimating the Effect of Intimate Partner Violence on Women’s Use of Contraception: A Systematic Review and Meta-Analysis
Source: PLoS One. 2015 Feb 18;10(2):e0118234. doi: 10.1371/journal.pone.0118234 (PMC4334227; doi:10.1371/journal.pone.0118234)
Supplement: S2 Table — (PDF) [file pone.0118234.s008.pdf]

Appendix Table S2. List of articles excluded after full text review with reasons for exclusion

	Article	Reason for exclusion
1.	Ackard DM, Eisenberg ME, Neumark-Sztainer D (2007) Long-term impact of adolescent dating violence on the behavioral and psychological health of male and female youth. The Journal of pediatrics 151: 476-481.	no measure of contraceptive use
2.	Ackerson LK, Subramanian S (2009) Intimate partner violence and death among infants and children in India. Pediatrics 124: e878-e889.	no longitudinal measure of IPV or contraceptive outcome
3.	Agurcia CA, Rickert VI, Berenson AB, Volk RJ, Wiemann CM (2001) The behavioral risks and life circumstances of adolescent mothers involved with older adult partners. Archives of Pediatrics and Adolescent Medicine 155: 822-830.	no measure of contraceptive use
4.	Ahmed S, Koenig MA, Stephenson R Effects of domestic violence on perinatal and early-childhood mortality: evidence from north India. Am J Public Health 96: 1423-1428.	no longitudinal measure of IPV or contraceptive outcome
5.	Alhusen JL, Lucea MB, Bullock L, Sharps P Intimate partner violence, substance use, and adverse neonatal outcomes among urban women. Journal of Pediatrics 163: 471-476.	no longitudinal measure of IPV or contraceptive outcome
6.	Alizadeh M, Samadirad B, Ravanshad Y, Khamenian Z, Azarfar A (2013) A case control study of married women under the violence during pregnancy in Tabriz. [Persian]. Iranian Journal of Obstetrics, Gynecology and Infertility 15: e8-Pe13.	no measure of contraceptive use

Appendix Table S2. List of articles excluded after full text review with reasons for exclusion

7.	Allsworth JE, Secura GM, Zhao QH, Madden T, Peipert JF (2013) The impact of emotional, physical, and sexual abuse on contraceptive method selection and discontinuation. American Journal of Public Health 103: 1857-1864.	Violence measure not limited to intimate partners or no violence measure
8.	Asling-Monemi K, Naved RT, Persson LA (2008) Violence against women and the risk of under-five mortality: analysis of community-based data from rural Bangladesh. Acta Paediatrica 97: 226-232.	no measure of contraceptive use
9.	Asling-Monemi K, Peña R, Ellsberg MC, Persson L (2003) Violence against women increases the risk of infant and child mortality: a case-referent study in Nicaragua. Bulletin of the World Health Organization 81: 10-16.	no measure of contraceptive use
10.	Audi CAF, Correa AMS, Latorre MD, Santiago SM (2008) The association between domestic violence during pregnancy and low birth weight or prematurity. [Portuguese, English]: Jornal de Pediatria. 84 (1) (pp 60-67), 2008. Date of Publication: January-February 2008.	no measure of contraceptive use
11.	Audi CAF, Segall-Correa AM, Santiago SM, Andrade MdGG, Perez-Escamila R (2008) Violence against pregnant women: prevalence and associated factors. Revista de Saude Publica 42: 877-885.	no measure of contraceptive use
12.	Baumgartner JN, Waszak Geary C, Tucker H, Wedderburn M (2009) The influence of early sexual debut and sexual violence on adolescent pregnancy: a matched case-control study in Jamaica. International perspectives on sexual & reproductive health 35: 21-28.	Violence measure not limited to intimate partners or no violence measure
13.	Berenson AB, Wiemann CM, Rowe TF, Rickert VI (1997) Inadequate weight gain among pregnant adolescents: risk factors and relationship to infant birth weight. American Journal of Obstetrics & Gynecology 176: 1220-1224; discussion 1224-1227.	Violence measure not limited to intimate partners or no violence measure

Appendix Table S2. List of articles excluded after full text review with reasons for exclusion

14.	Berenson AB, Wiemann CM, Wilkinson GS, Jones WA, Anderson GD (1994) Perinatal morbidity associated with violence experienced by pregnant women. American Journal of Obstetrics & Gynecology 170: 1760-1766; discussion 1766-1769.	no measure of contraceptive use
15.	Binka FN, Maude GH, Gyapong TM, Ross DA, Smith PG (1995) Risk factors for child mortality in Northern Ghana: A case-control study: International Journal of Epidemiology. 24 (1) (pp 127-135), 1995. Date of Publication: 1995.	no measure of contraceptive use
16.	Bloom T, Curry MA, Durham L (2007) Abuse and psychological stress as factors in high utilization of medical services during pregnancy. Issues in Mental Health Nursing 28: 849-866.	no measure of contraceptive use
17.	Born A (2012) Relationship violence and teenage parents. Journal of Infant, Child & Adolescent Psychotherapy 11: 368-375.	no longitudinal measure of IPV or contraceptive outcome
18.	Boufettal H, Majdi F, Belhouss A, Mahdaoui S, Noun M, et al. (2012) Domestic violence during pregnancy. [French]: Revue de Medecine Legale. 3 (2) (pp 72-78), 2012. Date of Publication: May 2012.	no measure of contraceptive use
19.	Bronfman MN (1995) Multimortalidad infantil y familia: dinámica, estructura y riesgo diferenciado. Infant multimortality and family: dynamics, structure and differentiated risk. Perinatol Reprod Hum 9: 11-28.	no measure of contraceptive use

Appendix Table S2. List of articles excluded after full text review with reasons for exclusion

20.	Browne A (1993) Violence against women by male partners: Prevalence, outcomes, and policy implications. American Psychologist 48: 1077.	no longitudinal measure of IPV or contraceptive outcome
21.	Buard V, Bergh Rvd, Tayler-Smith K, Godia P, Sobry A, et al. (2013) Characteristics, medical management and outcomes of survivors of sexual gender-based violence, Nairobi, Kenya. Public Health Action 3: 109-112.	no longitudinal measure of IPV or contraceptive outcome
22.	Budde MP, De Lange TE, Dekker GA, Chan A, Nguyen AMT (2007) Risk factors for placental abruption in a socio-economically disadvantaged region: Journal of Maternal-Fetal and Neonatal Medicine. 20 (9) (pp 687-693), 2007. Date of Publication: 2007.	no measure of contraceptive use
23.	Calandra N, Vázquez S, Berner E, Corral A, Bianculli C (1996) Embarazo adolescente. Adolescent pregnancy. Rev Soc Argent Ginecol Infanto Juvenil 3: 35-46.	no longitudinal measure of IPV or contraceptive outcome
24.	Campbell J, Torres S, Ryan J, King C, Campbell DW, et al. (1999) Physical and nonphysical partner abuse and other risk factors for low birth weight among full term and preterm babies: a multiethnic case-control study. American Journal of Epidemiology 150: 714-726.	no longitudinal measure of IPV or contraceptive outcome
25.	Campbell JC, Pugh LC, Campbell D, Visscher M (1995) The influence of abuse on pregnancy intention. Womens Health Issues 5: 214-223.	no measure of contraceptive use
26.	Campbell JC, Soeken KL (1999) Forced Sex and Intimate Partner Violence Effects on Women's Risk and Women's Health. Violence Against Women 5: 1017-1035.	no longitudinal measure of IPV or contraceptive outcome

Appendix Table S2. List of articles excluded after full text review with reasons for exclusion

27.	Chan RL, Martin SL (2009) Physical and sexual violence and subsequent contraception use among reproductive aged women. Contraception 80: 276-281.	no longitudinal measure of IPV or contraceptive outcome
28.	Chapagain M (2005) Masculine interest behind high prevalence of female contraceptive methods in rural Nepal. Australian Journal of Rural Health 13: 35-42.	no longitudinal measure of IPV or contraceptive outcome
29.	Chowdhary N, Patel V (2008) The effect of spousal violence on women's health: findings from the Stree Arogya Shodh in Goa, India. Journal of postgraduate medicine 54: 306.	no measure of contraceptive use
30.	Christofides NJ (2013) Adolescent pregnancy: Risk factors and consequences -- a longitudinal study in the Eastern Cape, South Africa. Dissertation Abstracts International: Section B: The Sciences and Engineering 73: No Pagination Specified.	Commentary, conference abstract, unpublished dissertation
31.	Clark CJ, Silverman J, Khalaf IA, Ra'ad BA, Al Sha'ar ZA, et al. (2008) Intimate partner violence and interference with women's efforts to avoid pregnancy in Jordan. Stud Fam Plann 39: 123-132.	no longitudinal measure of IPV or contraceptive outcome
32.	Clark LE, Allen RH, Goyal V, Raker C, Gottlieb AS (2013) Reproductive coercion and co-occurring intimate partner violence in obstetrics and gynecology patients. American Journal of Obstetrics & Gynecology.	no longitudinal measure of IPV or contraceptive outcome
33.	Coker AL, Sanderson M, Dong BL (2004) Partner violence during pregnancy and risk of adverse pregnancy outcomes. Paediatric and Perinatal Epidemiology 18: 260-269.	no measure of contraceptive use

Appendix Table S2. List of articles excluded after full text review with reasons for exclusion

34.	Coker Al, et al. (2000) Intimate partner violence and cervical neoplasia. Journal of women's health & gender-based medicine 9: 1015-1023.	no longitudinal measure of IPV or contraceptive outcome
35.	Connolly AM, Katz VL, Bash KL, McMahon MJ, Hansen WF (1997) Trauma and pregnancy. American Journal of Perinatology 14: 331-336.	no measure of contraceptive use
36.	Cottingham J, Garcia-Moreno C, Reis C (2008) Sexual and reproductive health in conflict areas: The imperative to address violence against women: BJOG: An International Journal of Obstetrics and Gynaecology. 115 (3) (pp 301-303), 2008. Date of Publication: February 2008.	no longitudinal measure of IPV or contraceptive outcome
37.	Covington DL, Hage M, Hall T, Mathis M (2001) Preterm delivery and the severity of violence during pregnancy: Journal of Reproductive Medicine for the Obstetrician and Gynecologist. 46 (12) (pp 1031-1039), 2001. Date of Publication: 2001.	no measure of contraceptive use
38.	Covington DL, Justason BJ, Wright LN (2001) Severity, manifestations, and consequences of violence among pregnant adolescents: Journal of Adolescent Health. 28 (1) (pp 55-61), 2001. Date of Publication: 2001.	no measure of contraceptive use
39.	Cox JE, Engelhart T, Snyder A, Forbes P, Arandia P, et al. (2012) Impact of domestic violence, relationships & contraceptive use on rapid repeat pregnancy in teen mothers. (Impact of Trauma on Teens: Building the Safety Net.). Journal of Adolescent Health 50: S78-S79.	Commentary, conference abstract, unpublished dissertation
40.	Cripe SM, Sanchez SE, Perales MT, Lam N, Garcia P (2008) Association of intimate partner physical and sexual violence with unintended pregnancy among pregnant women in Peru. International Journal of Gynecology and Obstetrics 100: 104-108.	no longitudinal measure of IPV or contraceptive outcome

Appendix Table S2. List of articles excluded after full text review with reasons for exclusion

41.	Curry MA, Doyle BA, Gilhooley J (1998) Abuse among pregnant adolescents: differences by developmental age. American Journal of Maternal Child Nursing 23: 144-150.	no measure of contraceptive use
42.	Curry MA, Perrin N, Wall E (1998) Effects of abuse on maternal complications and birth weight in adult and adolescent women. Obstetrics & Gynecology 92: 530-534.	Violence measure not limited to intimate partners or no violence measure
43.	D'Angelo DV, Gilbert BC, Rochat RW, Santelli JS, Herold JM (2004) Differences between mistimed and unwanted pregnancies among women who have live births. Perspectives on Sexual & Reproductive Health 36: 192-197.	no longitudinal measure of IPV or contraceptive outcome
44.	Dalal K, Andrews J, Dawad S (2011) CONTRACEPTION USE AND ASSOCIATIONS WITH INTIMATE PARTNER VIOLENCE AMONG WOMEN IN BANGLADESH. Journal of Biosocial Science: 1-12.	no longitudinal measure of IPV or contraceptive outcome
45.	Dallas CM (2013) Rapid repeat pregnancy among unmarried, African American adolescent parent couples. Western Journal of Nursing Research 35: 177-192.	no longitudinal measure of IPV or contraceptive outcome
46.	Debnath A, Roy N, Mazumder N (2013) Modern contraceptive behaviour of Indian women: Is spousal violence a constraint? International Journal of Sociology and Social Policy 33: 426-436.	no longitudinal measure of IPV or contraceptive outcome
47.	Deyessa N, Berhane Y, Emmelin M, Ellsberg MC, Kullgren G (2010) Joint effect of maternal depression and intimate partner violence on increased risk of child death in rural Ethiopia. Archives of Disease in Childhood 95: 771-775.	no measure of contraceptive use

Appendix Table S2. List of articles excluded after full text review with reasons for exclusion

48.	Dietz PM, Gazmaranian JA, Goodwin MM, Bruce FC, et al. (1997) Delayed entry into prenatal care: effect of physical violence. Obstetrics and Gynecology 90: 221-224.	no longitudinal measure of IPV or contraceptive outcome
49.	50. Dye TD, Tollivert NJ, Lee RV, Kenney CJ (1995) Violence, pregnancy and birth outcome in Appalachia. Paediatric and Perinatal Epidemiology 9: 35-47.	no measure of contraceptive use
50.	Eaton A (2008) Potential risk factors for HIV transmission in heterosexual HIV discordant couples: Intimate partner violence, antiretroviral therapies and concurrent sexual partners. Dissertation Abstracts International: Section B: The Sciences and Engineering 68: 5110.	Unpublished dissertation
51.	Ministerio de Salud Pública de Ecuador, Centro de Estudios de Población y Desarrollo, Codenpe, Unfpa, Naciones Unidas. (2006) Foro Permanente para las Cuestiones I Situación de salud de los pueblos indígenas en el Ecuador: encuesta demográfica y de salud materna e infantil-ENDEMAIN. Situation of health of the indigenous towns in the Ecuador: 112-112.	no measure of contraceptive use
52.	Edirne T, Can M, Kolusari A, Yildizhan R, Adali E, et al. Trends, characteristics, and outcomes of adolescent pregnancy in eastern Turkey. International Journal of Gynaecology & Obstetrics 110: 105-108.	no longitudinal measure of IPV or contraceptive outcome
53.	El Kady D, Gilbert WM, Xing G, Smith LH (2005) Maternal and neonatal outcomes of assaults during pregnancy. Obstetrics and Gynecology 105: 357-363.	no measure of contraceptive use
54.	Escribà-Agüir V, Romito P, Scrimin F, Molzan Turan J Are there differences in the impact of partner violence on reproductive health between postpartum women and women who had an elective abortion? Journal of Urban Health 89: 861-871.	no longitudinal measure of IPV or contraceptive outcome

Appendix Table S2. List of articles excluded after full text review with reasons for exclusion

55.	Fernandez FM, Krueger PM (1999) Domestic violence: effect on pregnancy outcome. Journal of the American Osteopathic Association 99: 254-256.	no measure of contraceptive use
56.	Fonseca SC, Coutinho ESF (2010) Risk factors for fetal mortality in a public maternity hospital in Rio de Janeiro, Brazil: a case-control study. [Portuguese]. Cadernos de Saude Publica 26: 240-252.	no measure of contraceptive use
57.	Fontenot HB, Fantasia HC (2011) Do women in abusive relationships have contraceptive control? Nursing for Women's Health 15: 239-243.	no longitudinal measure of IPV or contraceptive outcome
58.	Fox-Bartels T (2008) Intimate partner violence in pregnancy. On the Edge 14: 7p.	no longitudinal measure of IPV or contraceptive outcome
59.	Fried LE, Cabral H, Amaro H, Aschengrau A (2008) Lifetime and During Pregnancy Experience of Violence and the Risk of Low Birth Weight and Preterm Birth: Journal of Midwifery and Women's Health. 53 (6) (pp 522-528), 2008. Date of Publication: November 2008/December 2008.	no measure of contraceptive use
60.	Ganatra BR, Coyaji KJ, Rao VN (1998) Too far, too little, too late: a community-based case-control study of maternal mortality in rural west Maharashtra, India. Bulletin of the World Health Organization 76: 591-598.	no measure of contraceptive use

Appendix Table S2. List of articles excluded after full text review with reasons for exclusion

61.	Ganjiwale J (2012) Current health status of women in India - issues and challenges. Healthline, Journal of Indian Association of Preventive and Social Medicine 3: 60-63.	no longitudinal measure of IPV or contraceptive outcome
62.	Gao W, Paterson J, Carter S, Iusitini L (2007) Intimate partner violence and unplanned pregnancy in the Pacific Islands Families Study. International Journal of Gynaecology & Obstetrics 100: 109-115.	no longitudinal measure of IPV or contraceptive outcome
63.	66. Garoma S, Fantahun M, Worku A (2012) Maternal intimate partner violence victimization and under-five children mortality in Western Ethiopia: a case-control study. Journal of Tropical Pediatrics 58: 467-474.	no measure of contraceptive use
64.	Gavin AR, Lindhorst T, Lohr MJ (2011) The prevalence and correlates of depressive symptoms among adolescent mothers: results from a 17-year longitudinal study. Women Health 51: 525-545.	no measure of contraceptive use
65.	Gazmararian JA, Adams MM, Saltzman LE, Johnson CH, Bruce FC, et al. (1995) The relationship between pregnancy intendedness and physical violence in mothers of newborns. The PRAMS Working Group. Obstetrics & Gynecology 85: 1031-1038.	no longitudinal measure of IPV or contraceptive outcome
66.	Gee RE, Mitra N, Wan F, Chavkin DE, Long JA (2009) Power over parity: intimate partner violence and issues of fertility control. American Journal of Obstetrics & Gynecology 201: 148.e141-147.	no longitudinal measure of IPV or contraceptive outcome
67.	Gessessew A, Mesfin M (2004) Rape and related health problems in Adigrat Zonal Hospital, Tigray Region, Ethiopia. Ethiopian Journal of Health Development 18: 140-144.	no measure of contraceptive use

Appendix Table S2. List of articles excluded after full text review with reasons for exclusion

68.	Gielen AC, O'Campo PJ, Faden RR, Kass NE, Xue X (1994) Interpersonal conflict and physical violence during the childbearing year. Social Science & Medicine 39: 781-787.	no measure of contraceptive use
69.	Goodwin MM, Gazmararian JA, Johnson CH, Gilbert BC, Saltzman LE (2000) Pregnancy intendedness and physical abuse around the time of pregnancy: findings from the pregnancy risk assessment monitoring system, 1996-1997. PRAMS Working Group. Pregnancy Risk Assessment Monitoring System. Maternal & Child Health Journal 4: 85-92.	no longitudinal measure of IPV or contraceptive outcome
70.	Grimstad H, Schei B, Backe B, Jacobsen G (1997) Physical abuse and low birthweight: a case-control study. British Journal of Obstetrics & Gynaecology 104: 1281-1287.	no measure of contraceptive use
71.	Gutierres SE, Barr A (2003) The relationship between attitudes toward pregnancy and contraception use among drug users. Journal of Substance Abuse Treatment 24: 19-29.	Violence measure not limited to intimate partners or no violence measure
72.	Hamburger ME, Moore J, Koenig LJ, Vlahov D, et al. (2004) Persistence of inconsistent condom use: relation to abuse history and HIV serostatus. AIDS and Behavior 8: 333-344.	Violence measure not limited to intimate partners or no violence measure
73.	Harrykissoon SD, Rickert VI, Wiemann CM (2002) Prevalence and patterns of intimate partner violence among adolescent mothers during the postpartum period. Archives of Pediatrics & Adolescent Medicine 156: 325-330.	no measure of contraceptive use
74.	Holmes MM, Resnick HS, Kilpatrick DG, Best CL, Moore JG, et al. (1996) Rape-related pregnancy: Estimates and descriptive characteristics from a national sample of women: American Journal of Obstetrics and Gynecology. 175 (2) (pp 320-325), 1996. Date of Publication: 1996.	Violence measure not limited to intimate partners or no violence measure

Appendix Table S2. List of articles excluded after full text review with reasons for exclusion

75.	Huth-Bocks AC, Levendosky AA, Bogat GA (2002) The effects of domestic violence during pregnancy on maternal and infant health: Violence and Victims. 17 (2) (pp 169-185), 2002. Date of Publication: 2002.	no measure of contraceptive use
76.	Jacoby M, Gorenflo D, Wunderlich C, Eyler A (1999) Rapid repeat pregnancy and experiences of interpersonal violence among low-income adolescents. American Journal of Preventive Medicine 16: 318-321.	no measure of contraceptive use
77.	Jasinski JL (2001) Pregnancy and violence against women: An analysis of longitudinal data. Journal of Interpersonal Violence 16: 712-733.	no measure of contraceptive use
78.	Jejeebhoy SJ (1998) Associations between wife-beating and fetal and infant death: impressions from a survey in rural India. Stud Fam Plann 29: 300-308.	no longitudinal measure of IPV or contraceptive outcome
79.	Jewkes R, Vundule C, Maforah F, Jordaan E (2001) Relationship dynamics and teenage pregnancy in South Africa. Social Science & Medicine 52: 733-744.	no measure of contraceptive use
80.	Johnson PJ, Hellerstedt WL, Pirie PL (2002) Abuse history and nonoptimal prenatal weight gain. Public Health Reports 117: 148-156.	no measure of contraceptive use
81.	Jonsson U, Bohman H, Hjern A, von Knorring L, Paaren A, et al. (2011) Intimate relationships and childbearing after adolescent depression: a population-based 15 year follow-up study. Social Psychiatry and Psychiatric Epidemiology 46: 711-721.	no longitudinal measure of IPV or contraceptive outcome

Appendix Table S2. List of articles excluded after full text review with reasons for exclusion

82.	Kaye DK, Mirembe FM, Bantebya G, Johansson A, Ekstrom AM (2006) Domestic violence as risk factor for unwanted pregnancy and induced abortion in Mulago Hospital, Kampala, Uganda. Tropical Medicine & International Health 11: 90-101.	no measure of contraceptive use
83.	Kaye DK, Mirembe FM, Bantebya G, Johansson A, Ekstrom AM (2006) Domestic violence during pregnancy and risk of low birthweight and maternal complications: a prospective cohort study at Mulago Hospital, Uganda. Tropical medicine & international health : TM & IH 11: 1576-1584.	no measure of contraceptive use
84.	Kearney MH, Munro BH, Kelly U, Hawkins JW (2004) Health behaviors as mediators for the effect of partner abuse on infant birth weight. Nursing research 53.	no measure of contraceptive use
85.	Kearney MH, Haggerty LA, Munro BH, Hawkins JW (2003) Birth Outcomes and Maternal Morbidity in Abused Pregnant Women With Public Versus Private Health Insurance. Journal of Nursing Scholarship 35: 345-349.	no measure of contraceptive use
86.	Kiely M, El-Mohandes AA, Gantz MG, Chowdhury D, Thornberry JS, et al. (2011) Understanding the association of biomedical, psychosocial and behavioral risks with adverse pregnancy outcomes among African-Americans in Washington, DC. Maternal and Child Health Journal 15: S85-S95.	no measure of contraceptive use

Appendix Table S2. List of articles excluded after full text review with reasons for exclusion

87.	Kildea S, Stapleton H, Murphy R, Kosiak M, Gibbons K (2013) The maternal and neonatal outcomes for an urban Indigenous population compared with their non-Indigenous counterparts and a trend analysis over four triennia: BMC Pregnancy and Childbirth. 13 , 2013. Article Number: 167. Date of Publication: 30 Aug 2013.	no longitudinal measure of IPV or contraceptive outcome
88.	Kleijer ME, Dekker GA, Heard AR (2005) Risk factors for intrauterine growth restriction in a socio-economically disadvantaged region: Journal of Maternal-Fetal and Neonatal Medicine. 18 (1) (pp 23-30), 2005. Date of Publication: July 2005.	no measure of contraceptive use
89.	Leeners B, Stiller R, Block E, Gorres G, Rath W (2010) Pregnancy complications in women with childhood sexual abuse experiences. Journal of Psychosomatic Research 69: 503-510.	Violence measure not limited to intimate partners or no violence measure
90.	Lipsky S, et al. (2003) Impact of police-reported intimate partner violence during pregnancy on birth outcomes. Obstetrics and Gynecology 102: 557-564.	no measure of contraceptive use
91.	Lutz KF, et al. (2006) Double Binding, Abusive Intimate Partner Relationships, and Pregnancy. The Canadian journal of nursing research. Revue canadienne de recherche en sciences infirmières 38: 118-135.	no longitudinal measure of IPV or contraceptive outcome
92.	Macy RJ, Martin SL, Kupper LL, Casanueva C, Guo S (2007) Partner violence among women before, during, and after pregnancy: multiple opportunities for intervention. Womens Health Issues 17: 290-299.	no measure of contraceptive use
93.	Magill MK, Wilcox R (2007) Adolescent pregnancy and associated risks: not just a result of maternal age. American Family Physician 75: 1310-1311.	no longitudinal measure of IPV or contraceptive outcome

Appendix Table S2. List of articles excluded after full text review with reasons for exclusion

94.	Maughan B, Lindelow M (1997) Secular change in psychosocial risks: the case of teenage motherhood. Psychol Med 27: 1129-1144.	no measure of contraceptive use
95.	Mbassa Menick D, Dassa KS, Kenmogne JB, Abanda Ngon G (2009) Commercial sexual exploitation of minor girls. A multifocal, prospective study in Cameroon. [French]: Medecine Tropicale. 69 (1) (pp 91-96), 2009. Date of Publication: February 2009.	no longitudinal measure of IPV or contraceptive outcome
96.	McCleary-Sills JD (2012) Intimate partner violence and women's reproductive agency in Jordan. Dissertation Abstracts International: Section B: The Sciences and Engineering 72: 5922.	Commentary, conference abstract, unpublished dissertation
97.	McFarlane J, Campbell JC, Sharps P, Watson K (2002) Abuse during pregnancy and femicide: urgent implications for women's health. Obstetrics & Gynecology 100: 27-36.	no measure of contraceptive use
98.	McFarlane J, Parker B, Soeken K (1995) Abuse during pregnancy: frequency, severity, perpetrator, and risk factors of homicide. Public Health Nurs 12: 284-289.	no measure of contraceptive use
99.	McFarlane J, Parker B, Soeken K (1996) Physical abuse, smoking, and substance use during pregnancy: Prevalence, interrelationships, and effects on birth weight. Journal of Obstetric, Gynecologic, & Neonatal Nursing 25: 313-320.	no measure of contraceptive use
100.	McFarlane J, Soeken K (1999) Weight change of infants, age birth to 12 months, born to abused women. Pediatric Nursing 25: 19.	no measure of contraceptive use

Appendix Table S2. List of articles excluded after full text review with reasons for exclusion

101.	McFarlane J, et al. (1992) Assessing for abuse during pregnancy. Severity and frequency of injuries and associated entry into prenatal care. JAMA : the journal of the American Medical Association 267: 3176-3178.	no measure of contraceptive use
102.	McPherson M, Delva J, Cranford J (2007) A longitudinal investigation of intimate partner violence among mothers with mental illness. Psychiatric Services 58: 675-680.	no measure of contraceptive use
103.	Miller E, Jordan B, Levenson R, Silverman JG (2010) Reproductive coercion: connecting the dots between partner violence and unintended pregnancy. Contraception 81: 457-459.	no longitudinal measure of IPV or contraceptive outcome
104.	Miller E, McCauley H, Decker M, Tancredi DJ, Levenson R, et al. (2012) Partner violence and care-seeking patterns among female family planning clinic clients. (Impact of Trauma on Teens: Building the Safety Net.). Journal of Adolescent Health 50: S80-S81.	no longitudinal measure of IPV or contraceptive outcome
105.	Moore M (1999) Reproductive health and intimate partner violence. Family Planning Perspectives 31: 302-306.	no longitudinal measure of IPV or contraceptive outcome
106.	Moraes CL, et al. (2006) Gestational weight gain differentials in the presence of intimate partner violence. International Journal of Gynecology and Obstetrics 95: 254-260.	no measure of contraceptive use
107.	Nega A, Yemane B, Alemayehu W (2012) Wealth status, Mid Upper Arm Circumference (MUAC) and antenatal care (ANC) are determinants for low birth weight in Kersa, Ethiopia. PLoS ONE 7.	no measure of contraceptive use

Appendix Table S2. List of articles excluded after full text review with reasons for exclusion

108.	Nelson DB, Lepore SJ (2013) The role of stress, depression, and violence on unintended pregnancy among young urban women: Journal of Women's Health. 22 (8) (pp 673-680), 2013. Date of Publication: 01 Aug 2013.	no longitudinal measure of IPV or contraceptive outcome
109.	Noell J, Rohde P, Seeley J, Ochs L (2001) Childhood sexual abuse, adolescent sexual coercion and sexually transmitted infection acquisition among homeless female adolescents. Child Abuse & Neglect 25: 137-148.	Violence measure not limited to intimate partners or no violence measure
110.	Nunes MAA, Camey S, Ferri CP, Manzolli P, Manenti CN, et al. (2011) Violence during pregnancy and newborn outcomes: a cohort study in a disadvantaged population in Brazil. European Journal of Public Health 21: 92-97.	no measure of contraceptive use
111.	Nurul A, Saha SK, Abdur R, Ginneken JKv(2001) The effect of divorce on infant mortality in a remote area of Bangladesh. Journal of Biosocial Science 33: 271-278.	no measure of contraceptive use
112.	O'Campo P, Gielen AC, Faden RR, Kass N (1994) Verbal abuse and physical violence among a cohort of low-income pregnant women: Women's Health Issues. 4 (1) (pp 29-37), 1994. Date of Publication: 1994.	no measure of contraceptive use
113.	O'Donnell L, Agronick G, Duran R, Myint U, Stueve A (2009) Intimate partner violence among economically disadvantaged young adult women: Associations with adolescent risk-taking and pregnancy experiences. Perspectives on Sexual and Reproductive Health 41: 84-91.	no measure of contraceptive use
114.	Ordoñez M La situación de salud general y reproductiva de la población rural en Colombia. The situation of general and reproductive health of the rural population in Colombia. Estudio a profundidad de la ENDS-2000: 191-191.	no longitudinal measure of IPV or contraceptive outcome

Appendix Table S2. List of articles excluded after full text review with reasons for exclusion

115.	Otsea K (1999) Prioritizing reproductive health for refugees. Initiatives in Reproductive Health Policy 3: 1-3.	no longitudinal measure of IPV or contraceptive outcome
116.	Ouattara M, Sen P, Thomson M (1998) Forced marriage, forced sex: the perils of childhood for girls. Gender & Development 6: 27-33.	no longitudinal measure of IPV or contraceptive outcome
117.	Pak LL, Reece EA, Chan L (1998) Is adverse pregnancy outcome predictable after blunt abdominal trauma? American Journal of Obstetrics & Gynecology 179: 1140-1144.	no measure of contraceptive use
118.	Pallitto CC, Garcia-Moreno C, Jansen HA, Heise L, Ellsberg M, et al. (2013) Intimate partner violence, abortion, and unintended pregnancy: results from the WHO Multi-country Study on Women's Health and Domestic Violence. International Journal of Gynaecology and Obstetrics 120: 3-9.	no longitudinal measure of IPV or contraceptive outcome
119.	Panaretto K, Lee H, Mitchell M, Larkins S, Manessis V, et al. (2006) Risk factors for preterm, low birth weight and small for gestational age birth in urban Aboriginal and Torres Strait Islander women in Townsville. Australian & New Zealand Journal of Public Health 30: 163-170.	no measure of contraceptive use
120.	Parker B (2002) Childbearing experiences of abused Hispanic women: Journal of midwifery & women's health. 47 (5) (pp 401-402), 2002. Date of Publication: 2002 Sep-Oct.	no measure of contraceptive use

Appendix Table S2. List of articles excluded after full text review with reasons for exclusion

121.	Parker B, Chouaf K (2002) Intimate partner violence following pregnancy. Archives of Pediatrics & Adolescent Medicine 156: 313-314.	no measure of contraceptive use
122.	Parker B, McFarlane J, Soeken K (1994) Abuse during pregnancy: effects on maternal complications and birth weight in adult and teenage women. Obstetrics & Gynecology 84: 323-328.	no longitudinal measure of IPV or contraceptive outcome
123.	Parker B, et al (1993) Physical and emotional abuse in pregnancy: a comparison of adult and teenage women. Nursing research 42.	no measure of contraceptive use
124.	Patel SN (2013) Understanding hiv transmission risk in married hiv serodiscordant couples in Gujarat, India: The Positive Jeevan Saathi Study. Dissertation Abstracts International: Section B: The Sciences and Engineering 74: No Pagination Specified.	no longitudinal measure of IPV or contraceptive outcome
125.	Prosman G-J, Lo Fo Wong SH, Bulte E, Lagro-Janssen ALM (2012) Healthcare utilization by abused women: a case control study. European Journal of General Practice 18: 107-113.	no measure of contraceptive use
126.	Radhakrishna A, Gringle R, Greenslade F (1997) Adolescent women face triple jeopardy: unwanted pregnancy, HIV / AIDS and unsafe abortion. Women's Health Journal: 53-62.	no longitudinal measure of IPV or contraceptive outcome
127.	Ramashwar S (2009) South African women living in areas with high levels of sexual violence are at risk for HIV, early pregnancy. International perspectives on sexual & reproductive health 35: 211-212.	no longitudinal measure of IPV or contraceptive outcome

Appendix Table S2. List of articles excluded after full text review with reasons for exclusion

128.	Rico E, Fenn B, Abramsky T, Watts C (2011) Associations between maternal experiences of intimate partner violence and child nutrition and mortality: findings from Demographic and Health Surveys in Egypt, Honduras, Kenya, Malawi and Rwanda. Journal of Epidemiology & Community Health 65: 360-367.	no longitudinal measure of IPV or contraceptive outcome
129.	Roberts TA, Klein JD, Fisher S (2003) Longitudinal effect of intimate partner abuse on high-risk behavior among adolescents. Archives of Pediatrics & Adolescent Medicine 157: 875.	no measure of contraceptive use
130.	Rodrigues T, Rocha L, Barros H (2008) Physical abuse during pregnancy and preterm delivery. American Journal of Obstetrics & Gynecology 198: 171.e171-176.	no measure of contraceptive use
131.	Rosenberg J (2003) Abused women's children have an increased risk of dying before age five. International Family Planning Perspectives 29: [4].	no measure of contraceptive use
132.	Salam M, Alim M, Noguchi T (2006) Spousal Abuse Against Women and Its Consequences on Reproductive Health: A Study in the Urban Slums in Bangladesh. Maternal and Child Health Journal 10: 83-94.	no longitudinal measure of IPV or contraceptive outcome
133.	Salazar M, Valladares E, Öhman A, Högberg U (2009) Ending intimate partner violence after pregnancy: findings from a community-based longitudinal study in Nicaragua. BMC Public Health 9: 350.	no measure of contraceptive use
134.	Sanchez SE, Alva AV, Chang GD, Qiu C, Yanez D, et al. (2013) Risk of spontaneous preterm birth in relation to maternal exposure to intimate partner violence during pregnancy in Peru. Maternal and Child Health Journal 17: 485-492.	no measure of contraceptive use

Appendix Table S2. List of articles excluded after full text review with reasons for exclusion

135.	Sanchez SE, Qiu CF, Perales MT, Lam N, Garcia P, et al. (2008) Intimate partner violence (IPV) and preeclampsia among Peruvian women. European Journal of Obstetrics & Gynecology and Reproductive Biology 137: 50-55.	no measure of contraceptive use
136.	Saurel-Cubizolles MJ, Lelong N (2005) [Familial violence during pregnancy]. Journal de Gynecologie, Obstetrique et Biologie de la Reproduction 34: S47-53.	no measure of contraceptive use
137.	Shannon K, Csete J (2010) Violence, condom negotiation, and HIV/STI risk among sex workers: JAMA - Journal of the American Medical Association. 304 (5) (pp 573-574), 2010. Date of Publication: 04 Aug 2010.	no measure of contraceptive use
138.	Shroff MR, Griffiths PL, Suchindran C, Nagalla B, Vazir S, et al. (2011) Does maternal autonomy influence feeding practices and infant growth in rural India?: Social Science and Medicine. 73 (3) (pp 447-455), 2011. Date of Publication: August 2011.	no measure of contraceptive use
139.	Silverman JG, Decker MR, Cheng DM, Wirth K, Niranjan S, et al. (2011) Gender-based disparities in infant and child mortality based on maternal exposure to spousal violence: the heavy burden borne by Indian girls. Archives of Pediatrics & Adolescent Medicine 165: 22-27.	no measure of contraceptive use
140.	Sipsma HL, Magriples U, Divney A, Gordon D, Gabzdyl E, et al. (2013) Breastfeeding behavior among adolescents: initiation, duration, and exclusivity. Journal of Adolescent Health 53: 394-400.	no measure of contraceptive use
141.	Stephenson R, Koenig MA, Ahmed S (2006) Domestic violence and contraceptive adoption in Uttar Pradesh, India. Stud Fam Plann 37: 75-86.	no measure of contraceptive use

Appendix Table S2. List of articles excluded after full text review with reasons for exclusion

142.	Stephenson R, Koenig MA, Rajib A, Roy TK (2008) Domestic violence, contraceptive use, and unwanted pregnancy in rural India. Studies in Family Planning 39: 177-186.	Violence measure not limited to intimate partners or no violence measure
143.	Sun F, Tu X (2011) Effect of domestic violence on women reproductive health. [Chinese]. Journal of International Reproductive Health/Family Planning 30: 338-341.	no longitudinal measure of IPV or contraceptive outcome
144.	Swan H, O'Connell DJ (2012) The impact of intimate partner violence on women's condom negotiation efficacy. J Interpers Violence 27: 775-792.	no longitudinal measure of IPV or contraceptive outcome
145.	Taft AJ, Watson LF (2007) Termination of pregnancy: associations with partner violence and other factors in a national cohort of young Australian women. Australian & New Zealand Journal of Public Health 31: 135-142.	no measure of contraceptive use
146.	Talley PR (2002) Male violence and stress in pregnancy: Neuroendocrine parameters and length of gestation. Dissertation Abstracts International: Section B: The Sciences and Engineering 63: 2312.	no measure of contraceptive use
147.	Tan JCH, Gregor KV (2006) Violence against pregnant women in Northwestern Ontario. Annals of the New York Academy of Sciences 1087: 320-338.	no measure of contraceptive use
148.	Taneva D (2009) Undesired pregnancy and desired abortion. [Bulgarian]. Zdravna Politika i Menidzhm'nt / Health Policy and Management 1: 45-48.	no longitudinal measure of IPV or contraceptive outcome

Appendix Table S2. List of articles excluded after full text review with reasons for exclusion

149.	Tiwari A, Chan KL, Fong D, Leung WC, Brownridge DA, et al. (2008) The impact of psychological abuse by an intimate partner on the mental health of pregnant women. BJOG: An International Journal of Obstetrics & Gynaecology 115: 377-384.	no measure of contraceptive use
150.	Turner AN, Van Damme K, Jamieson DJ, Khan MR, Pettifor AE, et al. (2009) Predictors of adherent use of diaphragms and microbicide gel in a four-arm, randomized pilot study among female sex workers in Madagascar. Sexually Transmitted Diseases 36: 249-257.	Violence measure not limited to intimate partners or no violence measure
151.	Uscher-Pines L, Nelson DB (2010) Neighborhood and individual-level violence and unintended pregnancy. Journal of Urban Health: Bulletin of the New York Academy of Medicine 87: 677-687.	no longitudinal measure of IPV or contraceptive outcome
152.	Valentine JM, Rodriguez MA, Lapeyrouse LM, Zhang M (2011) Recent intimate partner violence as a prenatal predictor of maternal depression in the first year postpartum among Latinas. Archives of Women's Mental Health 14: 135-143.	no measure of contraceptive use
153.	Valladares E, Ellsberg M, Peña R, Högberg U, Persson L (2002) Physical partner abuse during pregnancy: a risk factor for low birth weight in Nicaragua. Obstetrics & Gynecology 100: 700-705.	no measure of contraceptive use
154.	VandeCastle M, Danna J, DeCoster E, Thomas T (1994) Physical violence during the 12 months preceding childbirth - Alaska, Maine, Oklahoma, and West Virginia, 1990-1991: Journal of the American Medical Association. 271 (15) (pp 1152-1153), 1994. Date of Publication: 1994.	no longitudinal measure of IPV or contraceptive outcome
155.	Varela ZMdV, Silva RM, Barroso MGT (1998) Diminsões do cotidiano: violência doméstica, saúde da mulher e desempenho no trabalho. Activities of daily living: women's health and domestic violence. 154-154.	Commentary, conference abstract, unpublished dissertation

Appendix Table S2. List of articles excluded after full text review with reasons for exclusion

156.	Vos T, et al (2006) Measuring the impact of intimate partner violence on the health of women in Victoria, Australia. Bulletin of the World Health Organization 84: 739-744.	no measure of contraceptive use
157.	Watson LF, Taft AJ, Lee C (2007) Associations of self-reported violence with age at menarche, first intercourse, and first birth among a national population sample of young Australian women. Womens Health Issues 17: 281-289.	no measure of contraceptive use
158.	Webster J, Chandler J, Battistutta D (1996) Pregnancy outcomes and health care use: effects of abuse. American Journal of Obstetrics & Gynecology 174: 760-767.	no measure of contraceptive use
159.	Wiemann CM, Agurcia CA, Berenson AB, Volk RJ, Rickert VI (2000) Pregnant adolescents: experiences and behaviors associated with physical assault by an intimate partner. Maternal and Child Health Journal 4: 93-101.	no measure of contraceptive use
160.	Williams C, McCloskey L (2006) Intimate partner violence and contraceptive use. [Unpublished] 2006. Presented at the Population Association of America, 2006 Annual Meeting, Los Angeles, California, March 30 - April 1, 2006. [21] p. p.	Commentary, conference abstract, unpublished dissertation
161.	Witte SS, Batsukh A, Chang M (2010) Sexual risk behaviors, alcohol abuse, and intimate partner violence among sex workers in Mongolia: implications for HIV prevention intervention development. (Special Issue: Global HIV prevention efforts with vulnerable women.). Journal of Prevention and Intervention in the Community 38: 89-103.	Violence measure not limited to intimate partners or no violence measure

Appendix Table S2. List of articles excluded after full text review with reasons for exclusion

162.	Woods SM, Melville JL, Guo Y, Fan MY, Gavin A (2010) Psychosocial stress during pregnancy: American Journal of Obstetrics and Gynecology. 202 (1) (pp 61.e1-61.e7), 2010. Date of Publication: January 2010.	no measure of contraceptive use
163.	Yimin C, Baohua K, Tieyan W, Xuejun H, Huan S, et al. (2001) Case-controlled study on relevant factors of adolescent sexual coercion in China: Contraception. 64 (2) (pp 77-80), 2001. Date of Publication: 2001.	Violence measure not limited to intimate partners or no violence measure
164.	Yimin C, Shouqing LI, Arzhu QU, Yuke Z, Jianhua W, et al. (2002) Sexual coercion among adolescent women seeking abortion in China: Journal of Adolescent Health. 31 (6) (pp 482-486), 2002. Date of Publication: 01 Dec 2002.	Violence measure not limited to intimate partners or no violence measure
165.	Zapata L Repercuciones de la violencia basada en el género sobre el embarazo. Repercussions of gender violence upon pregnancy. Rev Obstet Ginecol Venez 66: 21-27.	no measure of contraceptive use
166.	Zareen N, Majid N, Naqvi S, Saboohi S, Fatima H (2009) Effect of domestic violence on pregnancy outcome: Journal of the College of Physicians and Surgeons Pakistan. 19 (5) (pp 291-296), 2009. Date of Publication: May 2009.	no measure of contraceptive use
167.	Zunaira A, Zafar MI, Zahira B (2007) Impact assessment of wife battering on their reproductive health in rural areas of district Faisalabad. Science International 19: 313-317.	no measure of contraceptive use
