# Supplementary material for: Estimating the Effect of Intimate Partner Violence on Women’s Use of Contraception: A Systematic Review and Meta-Analysis
Source: PLoS One. 2015 Feb 18;10(2):e0118234. doi: 10.1371/journal.pone.0118234 (PMC4334227; doi:10.1371/journal.pone.0118234)
Supplement: S3 Table — (PDF) [file pone.0118234.s009.pdf]

Table S3. Summary of randomized controlled trials that examine the association between IPV and women contraceptive use.

Author, year	Population; country	Intervention arm	Control arm	Intimate partner	IPV	Outcome	OR (95% CI)	Probability of bias[a]	Qualitative summary of findings
Kacanek 2013 [43]	Subset of MIRA trial [71] participants ages 18-49 recruited from clinics. Limited to participants who answered questions on IPV at baseline and 12 month follow-up; South Africa, Zimbabwe	Diaphragm, lubricant gel, and condoms; product adherence and risk reduction counseling; HIV/STI testing and treatment. Women who report IPV referred to available services. N=950	Condoms; HIV/STI testing and treatment. Women who report IPV referred to available services. N=974	Person you most often had sex with in the last 3 months	Physical or sexual (any vs no IPV in past year)[b]	Sometimes/never vs always use of condoms in last 3 months[c]	1.66 (1.39, 1.98)	Low	Intervenes on IPV and outcome in intervention arm
Jones 2013 [44]	239 pregnant women age 18 or over who completed HIV counseling and testing and agreed to participate with their male partner, recruited from 12 antenatal clinics; South Africa	Group sessions to improve sexual negotiation, conflict resolution, STI/HIV prevention, PMTCT, use of male and female condoms, gender issues	Group health education sessions plus PMTCT	NR	NR	Consistent vs inconsistent condom use 6-8 weeks post baseline	NR	NA	No relevant effect measure (no measure of condom uptake by IPV status in either study arm). Intervenes on IPV and outcome
Wingood 2006 [39]	Subset of RCT of African-American adolescent girls [72] who reported their boyfriend forced sexual intercourse and reported having vaginal intercourse in past 6 months; USA	HIV prevention sessions (reduce HIV risk, enhance condom use and communication skills, build healthy relationships). N=73	General health promotion sessions. N=73	Boyfriend	Physical or sexual IPV	Consistent condom use in the last month	NR	NA	No relevant effect measure (restricted to girls who report baseline IPV, no additional IPV measure). Intervenes on IPV and outcome

OR, adjusted odds ratio; CI, confidence interval; MIRA, Methods for Improving Reproductive Health in Africa; HIV, human immuno-deficiency virus; STI, sexually transmitted infection; IPV, intimate partner violence; PMTCT, Prevention of maternal to child transmission; NR, Not reported; NA, Not applicable

[a]We do not evaluate bias in the estimate from the Jones and Wingood RCTs because these RCTs did not have an effect estimate relevant to our research question

[b]While the Kacanek study includes a more expansive definition of IPV that includes physical, sexual, emotional violence and fear of violence; we use the estimate that includes physical and sexual IPV for comparability given that no other study includes "fear of violence" as a form of IPV
