# Supplementary material for: Estimating the Effect of Intimate Partner Violence on Women’s Use of Contraception: A Systematic Review and Meta-Analysis
Source: PLoS One. 2015 Feb 18;10(2):e0118234. doi: 10.1371/journal.pone.0118234 (PMC4334227; doi:10.1371/journal.pone.0118234)
Supplement: S1 Text — (PDF) [file pone.0118234.s010.pdf]

Text S1 Ovid (Medline) search strategy.

1 (Millennium Development Goal* adj ("5" or "4" or "3")).mp. (264)
2 (MDG5 or MDG4 or MDG3 or MDG 3 or MDG 4 or MDG 5).mp. (451)
3 Maternal Mortality/ (7924)
4 ((postpartum or antepartum or postnatal or antenatal or perinatal or maternal) adj (death or mortal*)).mp. (22301)
5 Family Planning/ (22755)
6 Pregnancy, Unplanned/ (880)
7 Pregnancy, Unwanted/ (2228)
8 ((contra* or family planning or pill or method) adj (method or failure or method failure or discontinuation)).mp. (5234)
9 ((unplan* or unwant* or mistime* or wanted* or unintend* or intend*) adj pregnan*).mp. (5395)
10 (family planning or planned parenthood or birth control or reproductive health).mp. (53456)
11 (birth regulat* or population regulat* or fertility regulat* or birth spacing or pregnancy inter*).mp. (2754)
12 adolescent pregnancy/pc [Prevention] (974)
13 Contraception/ (16688)
14 Contraception Behavior/ (6369)
15 Birth Control/ (16688)
16 ((birth control or family planning or reproductive health) adj clinic).mp. (1168)
17 (intrauterine device* or intra-uterine device* or IUD*).mp. (13731)
18 (barrier method* or condom* or vaginal sponge* or cervical cap* or birth control* or birth control pill).mp. (22860)
19 (family planning* or family-planning*).mp. (44256)
20 ((covert or hidden) adj (contra* or pill or family planning or family planning method)).mp. (18)
21 ((female or woman or women) adj sterili*).mp. (3394)
22 exp Infant, Small for Gestational Age/ (5238)
23 Infant, Premature/et, mo, pc, px, sn [Etiology, Mortality, Prevention & Control, Psychology, Statistics & Numerical Data] (942)
24 Fetal Death/ep, et, mo, pc [Epidemiology, Etiology, Mortality, Prevention & Control] (8576)
25 Perinatal Mortality/ (776)
26 ((infant or perinatal) adj (mortality or morbidity or death)).mp. (44896)
27 ((preterm or premature) adj birth).mp. (13627)
28 (SGA or small for gestational age).mp. (9732)
29 Birth Intervals/ (1381)
30 ((birth or preg* or interpreg*) adj (spac* or interval)).mp. (1681)
31 ("unmet need for family planning" or "unmet need for contraception").tw. (227)
32 Rectovaginal Fistula/ep, et, pc [Epidemiology, Etiology, Prevention & Control] (520)
33 Pregnancy Outcome/ep, et, pc [Epidemiology, Etiology, Prevention & Control] (3613)
34 (fistula or preeclampsia or pre-eclampsia).mp. (111610)
35 Pre-Eclampsia/ep, et, pc [Epidemiology, Etiology, Prevention & Control] (4951)
36 prenatal care/ (20752)
37 postnatal care/ (3889)
38 ((ante-natal or pre-natal or post-natal or post nantal or ante natal or antenatal or postnatal) adj (care or clinic or health service)).mp. (10927)
39 (sex* adj (initiat* or debut)).mp. (1208)
40 (age adj3 ((sex* initiat* or first birth or first pregnancy or first intercour* or first sex* or sex* debut or first child* or initiation) adj2 sex)).mp. (199)
41 (skilled birth attend* or (skilled adj birth attend*)).mp. (313)
42 exp domestic violence/ (35705)

43 elder abuse/ (1898)

44 child abuse/ (17490)

45 42 not (43 or 44) (16439)

46 battered women/ (2347)

47 ((domestic or partner* or family or families or spouse or woman or women or female* or wife or wives or husband* or boyfriend* or girlfriend*) adj (abus* or batter* or violen* or beat* or batter*)).m_titl. (5552)

48 ((partner or relationship or domestic or spous* or date* or emotional or psychological) adj (abus* or violen*)).m_titl. (5187)

49 (intimate adj partner adj (abuse or victimi* or violence)).tw. (3378)

50 ((sexual or dating or physical or emotional) adj (violence or partner violence)).tw. (2952)

51 (adolescent sexual abuse or ((marital or spousal or date) adj rape)).tw. (256)

52 ((birth control or repro*) adj sabotage).tw. (3)

53 (comment or letter or editorial or conference abstract or news or newspaper article or patient education handout or case report).pt. (1489435)

54 systematic review.ti. (31913)

55 animals/ (5508483)

56 or/1-41 (297030)

57 or/45-52 (20533)

58 56 and 57 (1297)

59 58 not 53 (1256)

60 59 not 54 (1242)

61 60 not 55 (1242)
