# Supplementary material for: Estimating the Effect of Intimate Partner Violence on Women’s Use of Contraception: A Systematic Review and Meta-Analysis
Source: PLoS One. 2015 Feb 18;10(2):e0118234. doi: 10.1371/journal.pone.0118234 (PMC4334227; doi:10.1371/journal.pone.0118234)
Supplement: S2 Text — (PDF) [file pone.0118234.s011.pdf]

Systematic review protocol

TITLE OF THE REVIEW: Estimating the effect of intimate partner violence on women's reproductive health outcomes: A systematic review protocol

Review Team:
Lauren Maxwell, MPH; Danielle Zionts; Jacquelyn Campbell, PhD, RN, FAAN; Jeanne L. Alhusen, PhD, CRNP, RN; Karen Devries, BSc, PhD

Background:

In addition to being a human rights issue, intimate partner violence (IPV) is an important public health concern. The World Health Organization defines IPV as the self-report of physical or sexual violence by a current or former partner since the age of 15. (1) IPV is the most prevalent form of gender-based violence; the 2013 Global Burden of Disease Study estimates that 30% of women age 15 or over have experienced physical or sexual IPV. (2)

Prevalence studies from a number of countries indicate that IPV is associated with a constellation of women's reproductive health (RH) outcomes. Taking control of women's RH is one form of IPV. Women may be forced to have sex or to practice unprotected sex by their male partners and male partners may sabotage women's family planning (FP) to increase their female partner's dependency or to otherwise express their control over their partner's decision making.

Women's ability to control the timing, spacing, and number of their pregnancies is a critical health and human rights issue. Addressing the unmet need for FP is a key step to meeting Millennium Development Goals 3, 4 and 5 which include: promoting gender equity; reducing maternal and child mortality; and ensuring universal access to RH including FP and antenatal care.

While this analysis focuses on the association between IPV and women's RH outcomes, women's RH is intrinsically related to the health of their infants and children. Experience of IPV before or during pregnancy is associated with low birth weight infants, preterm birth, and increased risk of infant and child mortality in comparison to the children of mothers who do not experience IPV. (1)

Understanding how IPV affects women's use of FP and of maternity related health services in central to ensuring FP and pregnancy related RH services can better serve women who experience IPV. Understanding the global impact of IPV on women's RH is the first step towards building interventions that allow women who experience IPV to manage their fertility.

In this review, we will consider all author definitions of physical, sexual, and emotional IPV.

Rationale for this review:

Existing meta-analysis on the association between IPV and women's RH outcomes has focused on estimating the prevalence or prevalence odds of different RH outcomes for women who experience IPV. In this analysis we will limit our review to studies where women reported that IPV occurred before the outcome of interest. We will only include cohort or case-control studies where women's exposure to intimate partner violence was measured prior to the ascertainment of the RH outcome of interest.

The purpose of this review is to:

1) Understand the availability of longitudinal measures and the quality of those measures for of a number of different RH-related outcomes which findings from cross-sectional studies have indicated are associated with IPV.
2) Summarize existing evidence from cohort and case-control studies for the effect of IPV on women's RH outcomes. For RH outcomes where we have identified at least 3 relevant studies, we plan to estimate the odds or risk of each RH outcome in ever-partnered women of reproductive age who have experienced prior IPV as compared to women who have not experienced prior IPV.

REVIEW QUESTION:

How are RH-related outcomes for ever-partnered women and girls who have experienced IPV different from those of ever-partnered women and girls who have not experienced IPV?

Criteria for considering studies for the review:

Types of studies (designs):

We will consider studies of ever-partnered women and or girls of reproductive age (ages 15-49) that evaluate the association between respondents' exposure to IPV by a male partner and one or more of the following RH-related outcomes: unmet need for family planning; unwanted or mistimed pregnancy; birth spacing; age at first sex and age at first birth; use of prenatal or antenatal care; obstetric fistula; preeclampsia; maternal mortality; perinatal mortality; preterm birth; and small for gestational age. We will not consider induced abortion and low birth weight in the SR because these outcomes are part of an SR that is being conducted by the WHO.

Because we are interested in the causal relationship between IPV and women's RH outcomes, we will restrict our review to cohort or case-control studies. We will only include studies that compare ever-partnered women and girls who experienced IPV prior to the reproductive outcome of interest to ever-partnered women and girls who did not experience IPV prior to the outcome of interest. We will include all author definitions of women or girls' experience of IPV, whether the abuse is classified as physical, sexual, or emotional IPV. We will include multiple studies that use the same dataset if they examine different RH outcomes. For studies that examine the same RH outcome in the same dataset, we will include the study that we identify as being the highest quality during the data extraction and quality assessment phase of the review. Given the changes in access to and uptake of family planning over the last 30 years, we will include studies from 1980 onwards. We will not restrict studies by geographic location.

Types of participants:

All included studies must include study populations of ever-partnered women and/or girls. There are no geographical limitations placed on the location of study participants.

Types of exposures:

We will include studies where the exposure is defined as women or girls' experience of physical, sexual, or emotional IPV.

Outcome measures

1. Unmet need for family planning
2. Unwanted or mistimed pregnancy
3. FP uptake
4. FP method choice
5. Age at initiation of sexual intercourse
6. Age at first birth
7. Birth spacing
8. Use of antenatal or prenatal services
9. Birth attended by skilled health personnel
10. Obstetric fistula
11. Preeclampsia
12. Mortality related to pregnancy, abortion, or childbirth
13. Perinatal mortality
14. Preterm birth (birth before 37 weeks gestation)
15. Small for gestational age (weight below 10th percentile)

SEARCH METHODS

Because of the changes in women's access to and knowledge of family planning methods over time with the introduction of novel family planning methods such as the IUD and birth control pills, we will restrict our search to 1980 onwards.

Databases and other sources, time periods, search terms, language restrictions, unpublished data, etc.

Our search will include the following electronic databases:

Biomedical databases:
- PubMed (Medline)
- OvidSP (EMBASE, PsycINFO, CINAHL)
- Global Health Library (including LILACS, AFRO, EMRO, PAHO, WHOLIS, WPRO)
- POPLINE

In addition to the electronic database searches, we will seek to identify additional studies through:

- Reviewing reference lists from relevant reviews and studies for additional published and unpublished studies

Search strategy:

Medical Subject Headings (MeSH) and text based search terms for IPV and for RH outcomes were adapted from prior peer-reviewed literature and systematic reviews (SR) of intimate partner violence (3, 4) and RH outcomes. An information scientist reviewed the search strategy and pilot tested the search in Medline, EMBASE and PsycINFO with one of the authors (refer to Appendix 1 for Medline search terms).

REVIEW METHODS

Study selection methods:

This review will follow the PRISMA guidelines for the SR of non-randomized studies. (5) After finalizing the protocol, we will prospectively register the Systematic Review Protocol with the PROSPERO database of SRs (http://www.crd.york.ac.uk/prospero). One reviewer (LM) will conduct the search in coordination with an information scientist and will remove duplicate studies. One reviewer (LM) will identify additional studies through a review of related SRs. These additional studies will then be manually added to the EndNote database. Eligible studies must use a retrospective or prospective cohort or case-control study design where the exposure is measured prior to the outcome. In keeping with the Cochrane recommendations for the SR of non-randomized studies, we will review study design features, rather than study design labels to evaluate whether studies can be classified as cohort or case-control study designs. (6) Only studies that meet the inclusion criteria will be considered for the review. Two independent reviewers (LM and DZ) will screen the title and abstract of all studies identified using the search strategy to identify studies that meet the inclusion criteria. In cases of disagreement about study inclusion, the study will be classified as a relevant study. All eligible studies will be downloaded into EndNote. We will maintain an electronic file that lists excluded references by primary reason for exclusion. Because we are interested in comparing the numbers of longitudinal (cohort or case-study) versus cross-sectional measures of the association between IPV and RH related outcomes we will create a summary table that reports the number of cross-sectional studies for each outcome during the title-abstract screening process. We will not otherwise include cross-sectional studies in this SR.

Data extraction methods (including methods for resolving disagreements):

We will eliminate duplicate entries and obtain the full text of all studies that meet our initial inclusion criteria. Data will be extracted by two independent reviewers using a standardized, pre-piloted electronic data extraction form. In cases where the study authors did not include an estimate of prevalence or an effect measure, we will contact the authors for additional information or for individual level data. Discrepancies in data abstraction between assessors will be resolved through consensus.

Data items that will be collected:

The electronic data extraction form completed for each included study will record a description of the study location, design, sample characteristics and size, source of study participants, exposure and outcome definitions, measured confounders, 95% CI and the estimated measure of effect. Because of the importance of properly training interviewer's to ensure that survey participants feel safe enough to reveal IPV, (1) we will extract data on training the interviewers may have received in conducting IPV

interviews. We will also record data on the type and severity of violence and the ascertainment of the chronicity of violence and the reported RH outcomes.

Quality assessment methods and how quality data will be used:

We will assess study quality using a quality assessment checklist specific to IPV exposure and modified for each study design and a modified version of the Newcastle-Ottawa Scale, a quality assessment tool designed for use with cohort and case-control studies which evaluates the selection of groups into the study; comparability of exposed and unexposed groups; and the possibility for exposure or outcome misclassification. (7) The quality assessment tool will evaluate the exposure and outcome measures and whether these measures were made using validated tools. Because exposure to different forms of IPV are not necessarily correlated, (2) we will include an assessment of author's description of the distribution of the excluded form of IPV across exposure groups in studies that only measure one or two forms of IPV (physical, sexual, or emotional) in our evaluation of the appropriateness of the comparison group. For example, a study that compared women who experienced physical violence to women who did not, without measuring sexual or emotional IPV would be classified differently than a study that compared women who experienced physical IPV, without experiencing emotional and sexual IPV, to women who experienced neither physical, sexual, or emotional IPV.

The two reviewers will independently assess each study's potential for being affected by the most common forms of bias in observational studies: selection, misclassification, confounding, analytic, and attrition bias. Focusing on the assessment of selection and confounding bias, we will then classify studies as having low, moderate, or high probability of bias. Studies will only be classified as having low probability of bias if they are not obviously affected by the forms of bias being reviewed and if they use methods for causal inference in observational data to reduce the probability of unmeasured confounding such as propensity score matching. Studies subject to moderate bias will adjust for relevant confounders and have no obvious sources of selection or misclassification bias. We will resolve discrepancies in the estimated level of bias by consensus and will report our final assessment of the probability of bias for each study. We will include a detailed description of our bias analysis in the Systematic Review.

Selection bias

Selection bias is a major source of bias in non-randomized studies. We will evaluate selection bias by closely reviewing study inclusion and exclusion criteria.

Confounding bias

We will determine whether each study adjusted for the following important confounders of the association between IPV and RH outcome: maternal age; parity; respondent's education; experience of childhood sexual or physical abuse; refugee status; exposure conflict and to other forms of gender based violence; female genital mutilation; household income; urban/rural location. Given that prior experience of IPV may be related both to later experience of IPV and later RH outcomes, we will assess whether cohort studies adjust for experience of IPV at baseline.

Detection (misclassification) bias

Under-reporting of IPV is both difficult to evaluate and a pervasive issue in IPV-related research. Women who report IPV may experience an increase in IPV severity. Studies that do not interview participants alone or that use interviewers who have not been trained in interviewing survivors of IPV will likely have a greater problem with under-reporting of the exposure than studies that follow the WHO best practices for interviewing women about IPV. (1) Included studies will be subject to detection bias if survey interviewers' knowledge of women's experience of IPV modifies the way that they question women about their RH outcomes.

Attrition bias

Understanding whether participant attrition is related to the study exposure and/or outcome is important for assessing a study's level of bias. We will use reported response-rates (or the lack thereof) to assess attrition bias in cohort studies included in the analysis.

Main summary measures of effect

We will estimate odds ratios (ORs), risk ratios (RR), or hazard ratios (HR) and 95% confidence intervals of having experienced IPV by study type/RH outcome categorization. In studies that only measure sexual, physical, or emotional IPV; the unexposed group will include women who do not experience that specific form of IPV. In studies that measure both sexual and physical IPV, or that measure sexual, physical, and emotional IPV, the women who experienced either or any form of IPV will be compared to women who experience neither sexual nor physical IPV or who do not experience any form of sexual, physical, or emotional IPV. We will report effect measures separately for each RH outcome.

Data synthesis and meta-analysis methods:

We will conduct analysis to explore heterogeneity and, given adequate high and moderate quality studies, meta-analysis using Stata version 13.0 (StataCorp LP, College Station Texas).

Heterogeneity assessment:

We will use the I^2 statistic to qualitatively assess heterogeneity within subgroups defined by study quality, clinically important subgroups, and effect estimates. We will examine differences between estimates from studies conducted in high versus low and middle income countries. Additionally, we will examine heterogeneity in comparisons between population-based samples and select populations. Because we will only be able to compare estimates of the same RH outcome, we may have limited data with which to assess heterogeneity.

The I^2 statistic quantifies the proportion of variation across studies due to heterogeneity rather than chance. (8) Generalizing the Cochrane Handbook for Systematic Reviews recommendations, a I^2 statistic between 0% and 25% will be interpreted as an insignificant amount of heterogeneity; 25% to 75% will represent moderate heterogeneity; 75% to 100% will be considered as representing substantial heterogeneity. (9) Given sufficient studies for certain RH outcomes, we may also evaluate heterogeneity using the Q statistic.

Additional analyses:

Meta-analysis

We will compare measures of effect separately for cohort and case-control studies and RH outcome. If we have at least 3 high or medium quality studies for a given study category/RH outcome grouping, we will use random-effects meta-analysis to estimate a pooled measure of effect. For study/RH groupings with fewer than 3 studies, we will qualitatively describe the estimated prevalence or effect measure. We will weight study estimates using the inverse variance of the odds, risk, or hazard ratio. The OR, RR, or HR will compare the odds, risk, or hazard of experiencing the RH outcome among ever-partnered women and girls of reproductive age who have prior experience of IPV compared to ever-partnered women and girls of reproductive age who have not previously experienced IPV.

Subgroup analysis

Given that this is a SR of observational studies, we expect to find heterogeneous populations, designs, comparison groups, and outcomes. Given sufficient studies, we will conduct subgroup analysis of study type (cohort vs. case-control); study quality (high versus low or moderate), high income versus low-and-middle income countries; type of IPV (sexual, physical, or emotional); comparison group (ex. sexual and physical IPV versus no sexual and no physical IPV); and RH-related outcome measure. Given sufficient studies, we may also conduct separate subgroup analysis for women who experienced IPV during pregnancy given that the effect of IPV on infant outcomes is likely different between populations of only pregnancy women and populations of women of reproductive age. Given sufficient studies in a given study type/outcome category, we will use subgroup analysis to explore sources of heterogeneity and their likely influence on pooled measures of effect.

Women who disclose IPV may be subject to increased violence by their partners or other family members. In keeping with the WHO's guidelines for the protection of human subjects in domestic violence related research, women should either be interviewed privately about their experience of IPV or should complete the IPV-related survey questions themselves. (1) Given sufficient studies for a IPV/RH outcome related grouping, we will consider a subgroup analysis of studies that do and do not report using the WHO's guidelines for the protection of human subjects in domestic violence research.

Sensitivity analysis

We will conduct two sensitivity analyses. We will estimate the pooled OR by study design to assess differences in the pooled estimate between studies of different methodological quality and/or sample size. We undertook two sensitivity analyses in the stillbirth meta-analysis. First, we estimated the pooled OR by study design (cohort, cross-sectional). Maternal age is an important confounder of RH outcomes; we will assess sensitivity analysis by maternal age (under 30 versus 30 and over). This was considered important as various types of study designs may differ in methodological quality.

Assessment of publication bias

Because researchers are unlikely to publish research protocols for observational studies, they are likely subject to publication bias. The degree of probable publication bias will be assessed visually by reviewing the asymmetry of study estimates using funnel plots that compare log ORs were to their standard errors. If we have a sufficient number of studies to warrant a statistical significance test, we will use the Egger' test of funnel plot asymmetry at the p<0.10. (10)

References

1. Organization WH. Global and regional estimates of violence against women: prevalence and health effects of intimate partner violence and non-partner sexual violence. 2013.
2. Devries KM, Mak JY, Garcia-Moreno C, Petzold M, Child JC, Falder G, et al. The Global Prevalence of Intimate Partner Violence Against Women. Science. 2013. Epub 2013/06/22.
3. Friedman SH, Loue S. Incidence and prevalence of intimate partner violence by and against women with severe mental illness. J Womens Health (Larchmt). 2007;16(4):471-80. Epub 2007/05/25.
4. Ramsay J, Carter Y, Davidson L, Dunne D, Eldridge S, Feder G, et al. Advocacy interventions to reduce or eliminate violence and promote the physical and psychosocial well-being of women who experience intimate partner abuse. The Cochrane database of systematic reviews. 2009(3):CD005043. Epub 2009/07/10.
5. Moher D, Liberati A, Tetzlaff J, Altman DG. Preferred reporting items for systematic reviews and meta-analyses: the PRISMA statement. Annals of internal medicine. 2009;151(4):264-9.
6. Reeves BC, Deeks JJ, Higgins JP. 13 Including non-randomized studies. Cochrane handbook for systematic reviews of interventions. 2008;1:391.
7. Wells G, Shea B, O'connell D, Peterson J, Welch V, Losos M, et al. The Newcastle-Ottawa Scale (NOS) for assessing the quality of nonrandomised studies in meta-analyses. 2000.
8. Higgins JP, Thompson SG, Deeks JJ, Altman DG. Measuring inconsistency in meta-analyses. BMJ: British Medical Journal. 2003;327(7414):557.
9. Deeks J, Higgins J, Altman D. Chapter 9: Analysing data and undertaking meta-analyses. Cochrane Handbook for Systematic Reviews of Interventions Version 5.0. 1 (updated September 2008). The Cochrane Collaboration. Cochrane Handbook for Systematic Reviews of Interventions Version. 2008;5(0).
10. Peters JL, Sutton AJ, Jones DR, Abrams KR, Rushton L. Comparison of two methods to detect publication bias in meta-analysis. JAMA: the journal of the American Medical Association. 2006;295(6):676-80.
