# Supplementary material for: Estimating the Effect of Intimate Partner Violence on Women’s Use of Contraception: A Systematic Review and Meta-Analysis
Source: PLoS One. 2015 Feb 18;10(2):e0118234. doi: 10.1371/journal.pone.0118234 (PMC4334227; doi:10.1371/journal.pone.0118234)
Supplement: S3 Text — (PDF) [file pone.0118234.s012.pdf]

Methodological Quality Assessment of Observational Studies: Legend

Cohort		Cross-sectional		Case-control	
**External validity** ***(The extent to which the results of the study provide a correct basis for applicability to other circumstances)***					
**1**	Representativeness of the sample for the target population	**1**	Representativeness of the sample for the target population		
**2**	What percentage of selected individuals agreed to participate? **(in baseline cross-sectional where applicable)**	**2**	What percentage of selected individuals agreed to participate?	**1**	What percentage of cases and controls selected agreed to participate? **(in baseline cross-sectional where applicable)**
**Internal validity** ***(The extent to which systematic error, bias, is minimised in a study)***					
**Performance Bias** ***(Systematic differences in Measurement of Exposure)***					
**1**	How was circumcision status ascertained?	**1**	How was circumcision status ascertained?	**1**	How was circumcision status ascertained?
		**2**	Were assessors of circumcision status blindedto HIV status?	**2**	Were assessors of circumcision status blinded to HIV status?
				**3**	Was the same method of ascertainment of circumcision status used for cases and controls?
**Detection Bias** ***(Systematic differences in outcome assessment)***					
**2**	How was HIV status ascertained?	**3**	How was HIV status ascertained?	**4**	How was HIV status ascertained?
**3**	How was HIV status confirmed?	**4**	How was HIV status confirmed?	**5**	How was HIV status confirmed?
**4**	Were assessors of HIV status blinded to circumcision status?	**5**	Were assessors of HIV status blinded to circumcision status?	**6**	Were assessors of HIV status blinded to circumcision status?
**Attrition bias** ***(Systematic differences in follow-up)***					
**5**	Were both groups followed-up for the same amount of time?				
**6**	Adequacy of follow-up of cohorts percentage of participants included in the final analysis)	**6**	Percentage of participants included in the final analysis?	**7**	Percentage of participants included in the final analysis: percentage HIV positive participants (cases)? Percentage HIV negative participants (controls)?
**Selection Bias / Control of confounding** ***(Systematic differences in comparison groups)***					
**7**	Demonstration that no person was HIV positive at the start of the study			**8**	How were the cases selected?
				**9**	How was the control group selected?
