# Supplementary material for: Estimating the Effect of Intimate Partner Violence on Women’s Use of Contraception: A Systematic Review and Meta-Analysis
Source: PLoS One. 2015 Feb 18;10(2):e0118234. doi: 10.1371/journal.pone.0118234 (PMC4334227; doi:10.1371/journal.pone.0118234)

The Cochrane Collaboration's tool for assessing risk of bias

Domain	Description	Review authors' judgement
**Sequence generation.**	Describe the method used to generate the allocation sequence in sufficient detail to allow an assessment of whether it should produce comparable groups.	Was the allocation sequence adequately generated?
**Allocation concealment.**	Describe the method used to conceal the allocation sequence in sufficient detail to determine whether intervention allocations could have been foreseen in advance of, or during, enrolment.	Was allocation adequately concealed?
**Blinding of participants, personnel and outcome assessors** *Assessments should be made for each main outcome (or class of outcomes).*	Describe all measures used, if any, to blind study participants and personnel from knowledge of which intervention a participant received. Provide any information relating to whether the intended blinding was effective.	Was knowledge of the allocated intervention adequately prevented during the study?
**Incomplete outcome data** *Assessments should be made for each main outcome (or class of outcomes).*	Describe the completeness of outcome data for each main outcome, including attrition and exclusions from the analysis. State whether attrition and exclusions were reported, the numbers in each intervention group (compared with total randomized participants), reasons for attrition/exclusions where reported, and any re-inclusions in analyses performed by the review authors.	Were incomplete outcome data adequately addressed?
**Selective outcome reporting.**	State how the possibility of selective outcome reporting was examined by the review authors, and what was found.	Are reports of the study free of suggestion of selective outcome reporting?
**Other sources of bias.**	State any important concerns about bias not addressed in the other domains in the tool.<br>If particular questions/entries were pre-specified in the review's protocol, responses should be provided for each question/entry.	Was the study apparently free of other problems that could put it at a high risk of bias?

Criteria for judging risk of bias in the 'Risk of bias' assessment tool

<table>
<tr><td colspan="2"><h2>SEQUENCE GENERATION</h2>Was the allocation sequence adequately generated? [Short form: Adequate sequence generation?]</td></tr>
<tr><td>Criteria for a judgement of 'YES' (i.e. low risk of bias).</td><td>The investigators describe a random component in the sequence generation process such as:
Referring to a random number table;
Using a computer random number generator;
Coin tossing;
Shuffling cards or envelopes;
Throwing dice;
Drawing of lots;
Minimization*.
*Minimization may be implemented without a random element, and this is considered to be equivalent to being random.</td></tr>
<tr><td>Criteria for the judgement of 'NO' (i.e. high risk of bias).</td><td>The investigators describe a non-random component in the sequence generation process. Usually, the description would involve some systematic, non-random approach, for example:
Sequence generated by odd or even date of birth;
Sequence generated by some rule based on date (or day) of admission;
Sequence generated by some rule based on hospital or clinic record number.
Other non-random approaches happen much less frequently than the systematic approaches mentioned above and tend to be obvious. They usually involve judgement or some method of non-random categorization of participants, for example:
Allocation by judgement of the clinician;
Allocation by preference of the participant;
Allocation based on the results of a laboratory test or a series of tests;
Allocation by availability of the intervention.</td></tr>
<tr><td>Criteria for the judgement of 'UNCLEAR' (uncertain risk of bias).</td><td>Insufficient information about the sequence generation process to permit judgement of 'Yes' or 'No'.</td></tr>
<tr><td colspan="2"><h2>ALLOCATION CONCEALMENT</h2>Was allocation adequately concealed? [Short form: Allocation concealment?]</td></tr>
<tr><td>Criteria for a judgement of 'YES' (i.e. low risk of bias).</td><td>Participants and investigators enrolling participants could not foresee assignment because one of the following, or an equivalent method, was used to conceal allocation:</td></tr>
</table>

	• Central allocation (including telephone, web-based and pharmacy-controlled randomization);<br>• Sequentially numbered drug containers of identical appearance;<br>• Sequentially numbered, opaque, sealed envelopes.
Criteria for the judgement of 'NO' (i.e. high risk of bias).	Participants or investigators enrolling participants could possibly foresee assignments and thus introduce selection bias, such as allocation based on:<br>• Using an open random allocation schedule (e.g. a list of random numbers);<br>• Assignment envelopes were used without appropriate safeguards (e.g. if envelopes were unsealed or nonopaque or not sequentially numbered);<br>• Alternation or rotation;<br>• Date of birth;<br>• Case record number;<br>• Any other explicitly unconcealed procedure.
Criteria for the judgement of 'UNCLEAR' (uncertain risk of bias).	Insufficient information to permit judgement of 'Yes' or 'No'. This is usually the case if the method of concealment is not described or not described in sufficient detail to allow a definite judgement – for example if the use of assignment envelopes is described, but it remains unclear whether envelopes were sequentially numbered, opaque and sealed.

BLINDING OF PARTICIPANTS, PERSONNEL AND OUTCOME ASSESSORS

Was knowledge of the allocated interventions adequately prevented during the study? [Short form: *Blinding*?]

Criteria for a judgement of 'YES' (i.e. low risk of bias).	Any one of the following:<br>• No blinding, but the review authors judge that the outcome and the outcome measurement are not likely to be influenced by lack of blinding;<br>• Blinding of participants and key study personnel ensured, and unlikely that the blinding could have been broken;<br>• Either participants or some key study personnel were not blinded, but outcome assessment was blinded and the non-blinding of others unlikely to introduce bias.
Criteria for the judgement of 'NO' (i.e. high risk of bias).	Any one of the following:<br>• No blinding or incomplete blinding, and the outcome or outcome measurement is likely to be influenced by lack of blinding;<br>• Blinding of key study participants and personnel attempted, but likely that the blinding could have been broken;<br>• Either participants or some key study personnel were not blinded, and the non-blinding of others likely to introduce bias.
Criteria for the judgement of 'UNCLEAR' (uncertain risk of bias).	Any one of the following:<br>• Insufficient information to permit judgement of 'Yes' or 'No';<br>• The study did not address this outcome.

INCOMPLETE OUTCOME DATA

Were incomplete outcome data adequately addressed? [Short form: *Incomplete outcome data addressed*?]

Criteria for a judgement of 'YES' (i.e. low risk of bias).	Any one of the following:<br>• No missing outcome data;<br>• Reasons for missing outcome data unlikely to be related to true outcome (for survival data, censoring unlikely to be introducing bias);<br>• Missing outcome data balanced in numbers across intervention groups, with similar reasons for missing data across groups;<br>• For dichotomous outcome data, the proportion of missing outcomes compared with observed event risk not enough to have a clinically relevant impact on the intervention effect estimate;<br>• For continuous outcome data, plausible effect size (difference in means or standardized difference in means) among missing outcomes not enough to have a clinically relevant impact on observed effect size;<br>• Missing data have been imputed using appropriate methods.
Criteria for the judgement of 'NO' (i.e. high risk of bias).	Any one of the following:<br>• Reason for missing outcome data likely to be related to true outcome, with either imbalance in numbers or reasons for missing data across intervention groups;<br>• For dichotomous outcome data, the proportion of missing outcomes compared with observed event risk enough to induce clinically relevant bias in intervention effect estimate;<br>• For continuous outcome data, plausible effect size (difference in means or standardized difference in means) among missing outcomes enough to induce clinically relevant bias in observed effect size;<br>• 'As-treated' analysis done with substantial departure of the intervention received from that assigned at randomization;<br>• Potentially inappropriate application of simple imputation.
Criteria for the judgement of 'UNCLEAR' (uncertain risk of bias).	Any one of the following:<br>• Insufficient reporting of attrition/exclusions to permit judgement of 'Yes' or 'No' (e.g. number randomized not stated, no reasons for missing data provided);<br>• The study did not address this outcome.

SELECTIVE OUTCOME REPORTING

Are reports of the study free of suggestion of selective outcome reporting? [Short form: *Free of selective reporting*?]

Criteria for a judgement of 'YES' (i.e. low risk of bias).	Any of the following:<br>• The study protocol is available and all of the study's pre-specified (primary and secondary) outcomes that are of interest in the review have been reported in the pre-specified way;

	• The study protocol is not available but it is clear that the published reports include all expected outcomes, including those that were pre-specified (convincing text of this nature may be uncommon).
Criteria for the judgement of 'NO' (i.e. high risk of bias).	Any one of the following:<br>• Not all of the study's pre-specified primary outcomes have been reported;<br>• One or more primary outcomes is reported using measurements, analysis methods or subsets of the data (e.g. subscales) that were not pre-specified;<br>• One or more reported primary outcomes were not pre-specified (unless clear justification for their reporting is provided, such as an unexpected adverse effect);<br>• One or more outcomes of interest in the review are reported incompletely so that they cannot be entered in a meta-analysis;<br>• The study report fails to include results for a key outcome that would be expected to have been reported for such a study.
Criteria for the judgement of 'UNCLEAR' (uncertain risk of bias).	Insufficient information to permit judgement of 'Yes' or 'No'. It is likely that the majority of studies will fall into this category.

OTHER POTENTIAL THREATS TO VALIDITY

Was the study apparently free of other problems that could put it at a risk of bias? [Short form: *Free of other bias*?]

Criteria for a judgement of 'YES' (i.e. low risk of bias).	The study appears to be free of other sources of bias.
Criteria for the judgement of 'NO' (i.e. high risk of bias).	There is at least one important risk of bias. For example, the study:<br>• Had a potential source of bias related to the specific study design used; or<br>• Stopped early due to some data-dependent process (including a formal-stopping rule); or<br>• Had extreme baseline imbalance; or<br>• Has been claimed to have been fraudulent; or<br>• Had some other problem.
Criteria for the judgement of 'UNCLEAR' (uncertain risk of bias).	There may be a risk of bias, but there is either:<br>• Insufficient information to assess whether an important risk of bias exists; or<br>• Insufficient rationale or evidence that an identified problem will introduce bias.

Example of a 'Risk of bias' table for a single study (fictional)

Entry	Judgement	Description
Adequate sequence generation?	Yes.	Quote: "patients were randomly allocated." Comment: Probably done, since earlier reports from the same investigators clearly describe use of random sequences (Cartwright 1980).
Allocation concealment?	No.	Quote: "...using a table of random numbers." Comment: Probably not done.
Blinding? (Patient-reported outcomes)	Yes.	Quote: "double blind, double dummy"; "High and low dose tablets or capsules were indistinguishable in all aspects of their outward appearance. For each drug an identically matched placebo was available (the success of blinding was evaluated by examining the drugs before distribution)." Comment: Probably done.
Blinding? (Mortality)	Yes.	Obtained from medical records; review authors do not believe this will introduce bias.
Incomplete outcome data addressed? (Short-term outcomes (2-6 wks))	No.	4 weeks: 17/110 missing from intervention group (9 due to 'lack of efficacy'); 7/113 missing from control group (2 due to 'lack of efficacy').
Incomplete outcome data addressed? (Longer-term outcomes (>6 wks))	No.	12 weeks: 31/110 missing from intervention group; 18/113 missing from control group. Reasons differ across groups.
Free of selective reporting?	No.	Three rating scales for cognition listed in Methods, but only one reported.
Free of other bias?	No.	Trial stopped early due to apparent benefit.

Example of a 'Risk of bias graph' figure

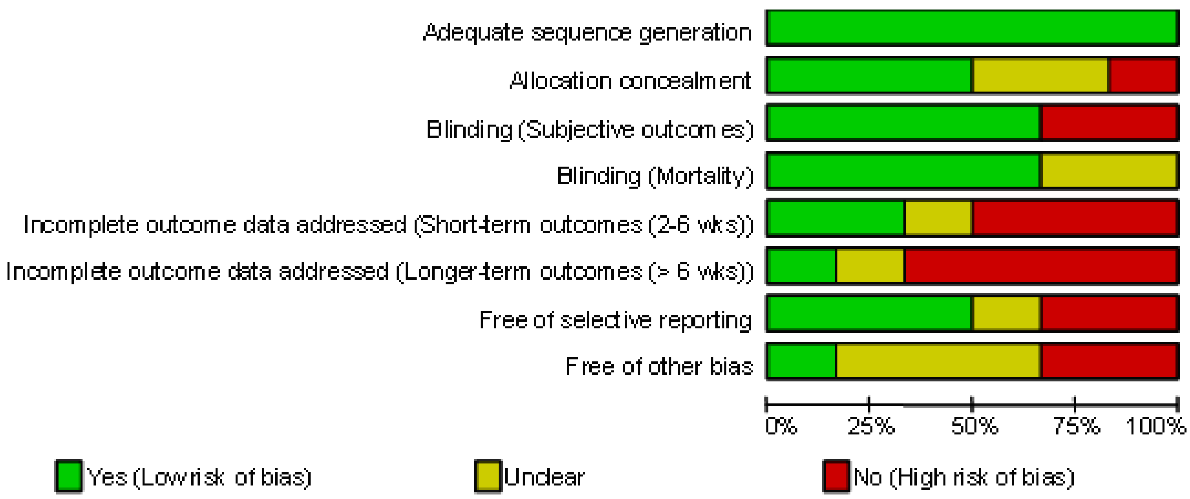

Supplement: S4 Text — (PDF) [file pone.0118234.s013.pdf]
